# Risk of arrhythmias following COVID-19: nationwide self-controlled case series and matched cohort study

**DOI:** 10.1093/ehjopen/oead120

**Published:** 2023-11-21

**Authors:** Ioannis Katsoularis, Hanna Jerndal, Sebastian Kalucza, Krister Lindmark, Osvaldo Fonseca-Rodríguez, Anne-Marie Fors Connolly

**Affiliations:** Department of Public Health and Clinical Medicine, Umeå University, 90187 Umeå, Sweden; Department of Clinical Microbiology, Umeå University, 90187 Umeå, Sweden; Department of Clinical Microbiology, Umeå University, 90187 Umeå, Sweden; Department of Public Health and Clinical Medicine, Umeå University, 90187 Umeå, Sweden; Department of Clinical Sciences, Karolinska Institutet, 17177 Stockholm, Sweden; Department of Clinical Microbiology, Umeå University, 90187 Umeå, Sweden; Department of Clinical Microbiology, Umeå University, 90187 Umeå, Sweden

**Keywords:** COVID-19, Arrhythmias, Self-controlled case series study

## Abstract

**Aims:**

COVID-19 increases the risk of cardiovascular disease, especially thrombotic complications. There is less knowledge on the risk of arrhythmias after COVID-19. In this study, we aimed to quantify the risk of arrhythmias following COVID-19.

**Methods and results:**

This study was based on national register data on all individuals in Sweden who tested positive for SARS-CoV-2 between 1 February 2020 and 25 May 2021. The outcome was incident cardiac arrhythmias, defined as international classification of diseases (10th revision) codes in the registers as follows: atrial arrhythmias; paroxysmal supraventricular tachycardias; bradyarrhythmias; and ventricular arrhythmias. A self-controlled case series study and a matched cohort study, using conditional Poisson regression, were performed to determine the incidence rate ratio and risk ratio, respectively, for an arrhythmia event following COVID-19.A total of 1 057 174 exposed (COVID-19) individuals were included in the study as well as 4 074 844 matched unexposed individuals. The incidence rate ratio of atrial tachycardias, paroxysmal supraventricular tachycardias, and bradyarrhythmias was significantly increased up to 60, 180, and 14 days after COVID-19, respectively. In the matched cohort study, the risk ratio during Days 1–30 following COVID-19/index date was 12.28 (10.79–13.96), 5.26 (3.74–7.42), and 3.36 (2.42–4.68), respectively, for the three outcomes. The risks were generally higher in older individuals, in unvaccinated individuals, and in individuals with more severe COVID-19. The risk of ventricular arrhythmias was not increased.

**Conclusion:**

There is an increased risk of cardiac arrhythmias following COVID-19, and particularly increased in elderly vulnerable individuals, as well as in individuals with severe COVID-19.

## Graphical Abstract

**Graphical Abstract:**
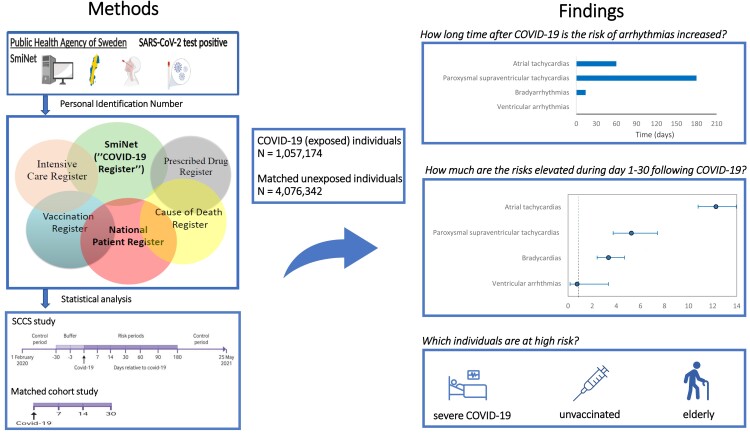
Overview of the study methods and main findings.

## Introduction

Coronavirus disease 2019 (COVID-19) is caused by infection with severe acute respiratory coronavirus 2 (SARS-CoV-2). Even though initially thought to be primarily a respiratory illness,^1^ COVID-19 is a multiorgan disease^2^ and increases the risk of cardiovascular disease both in the short-^3^ and long-terms.^4^ We previously identified COVID-19 as a risk factor for myocardial infarction, stroke,^5^ venous thromboembolism, and bleeding,^6^ which are known risk factors for cardiac arrhythmias. Arrhythmias, in turn, are associated with worse prognosis and quality of life both in COVID-19^7^ and non-COVID-19,^8^ by increasing the risk of morbidity, e.g. stroke, and mortality.

Less evidence exists on the risk of arrhythmias after COVID-19. Previous studies included mainly hospitalized patients with more severe COVID-19 and had a narrow selection of arrhythmia outcomes.^9,10^ There are no nationwide studies published on this topic including all COVID-19 cases registered in the country.

In this study, the aim was to determine the risk and duration of increased risk of cardiac arrhythmias following COVID-19, including all individuals tested positive for SARS-CoV-2 in Sweden.

## Methods

### Data source

All individuals with a positive SARS-CoV-2 test in Sweden have been reported to the communicable disease surveillance system SmiNet (Public Health Agency of Sweden). All individuals with a laboratory-verified positive test for SARS-CoV-2 infection between 1 February 2020 and 25 May 2021 were included in the study. Unexposed individuals were individuals without a laboratory-verified positive SARS-CoV-2 test. Unfortunately, we did not have access to data on individuals with negative SARS-COV-2 tests, as such individuals have not been registered in SmiNet or any other national register. Statistics Sweden identified four unexposed individuals for each COVID-19 individual matched on age (exact matching on year), sex, and county of residence, to minimize the differences between the cohorts.

Using the Personal Identification Numbers (PINs) of each COVID-19 and unexposed individual, we cross-linked the PINs with the SmiNet database, the National Inpatient Register (IPR, COVID-19 cases: 1987–2021, unexposed individuals: 1997–2021); the National Outpatient Register (OPR, 1997 to 2021); the National Cause of Death Register (2020–2021); and the National Prescribed Drug Register (2015–2021) and the National Vaccination Register (2021) from the Swedish National Board of Health and Welfare as well as the Swedish Intensive Care Register (2020–2021).

### Exposure

Exposure was defined as a positive laboratory-verified SARS-CoV-2 test. COVID-19 individuals were assigned a COVID-19 date defined as the earliest date available from the SmiNet according to: date of disease onset; sample date; diagnosis date; or date of report to SmiNet. Unexposed individuals were assigned an index date, which was the corresponding COVID-19 date for the matched SARS-CoV-2 test positive individuals.

### Outcomes

Outcome was an arrhythmia event defined as International Classification of Diseases (ICD-9 and ICD-10) diagnosis or intervention codes for arrhythmias in the National Outpatient or Inpatient Registers. The outcomes were divided into four different subgroups as follows: atrial arrhythmias (ATs) including atrial fibrillation and atrial flutter; paroxysmal supraventricular tachycardias (PSVTs) including AV-node re-entrant tachycardia; atrioventricular re-entrant tachycardia, Wolf–Parkinson–White syndrome, and ectopic atrial tachycardia; bradyarrhythmias (BAs) including sick sinus syndrome and high-grade AV-blocks II and III; and ventricular arrhythmias (VAs) including ventricular tachycardia, ventricular flutter, and ventricular fibrillation (see supplementary material online, table  *S1*). Diagnosis codes for sudden cardiac death were not included in the outcome VA, since the diagnosis of sudden cardiac death is caused by many different diagnoses, besides VAs. Individuals with prior events of the same type before the study periods were excluded; thus, only first events during the study period were included. All individuals with an outcome were assigned an event date, defined as the date of hospital admission or contact with an outpatient clinic due to arrhythmia as a main or contributing cause of contact.

### Statistical analyses

We used two different methodologies to estimate the relative risk of an arrhythmia event as follows: the self-controlled case series (SCCS) method and the matched cohort study (MCS). In addition, we have calculated the absolute risks (ARs) of events during the period 30 days following COVID-19 date, defined as the proportion of individuals who had an event. The ARs were calculated separately for the exposed and unexposed cohorts. There were no missing data in our analyses.

### Self-controlled case series study

**Figure 1:**
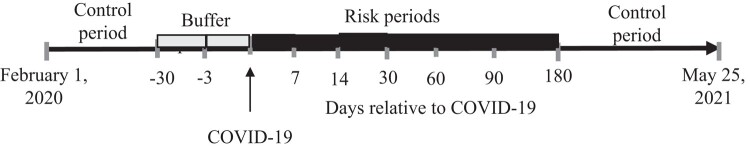
Flow diagram of study.

The SCCS method was used to investigate the temporal relationship between COVID-19 and arrhythmias, by calculating the incidence rate ratio (IRR) of a first arrhythmia event in risk periods following COVID-19. The SCCS method^11^ is a conditional Poisson regression method that compares intra-person incidence rates of an outcome (for example arrhythmias) in different time periods relative to an exposure (for example COVID-19). The analyses are automatically adjusted for time-fixed (measured or unmeasured) confounders, such as sex and chronic conditions. Only individuals who are both exposed (COVID-19) and have an outcome (arrhythmia) during the study period are included in the analyses. The study period (1 February 2020 to 25 May 2021) was divided into different risk periods relative to COVID-19 date (Days 0, 1–7, 8–14, 15–30, 31–60, 61–90, and 91–180 post-COVID-19). We noticed an increased number of events at Day 0 (COVID-19 date), as in previous studies,^5,6^ indicating likelihood for test bias.^12^ Therefore, Day 0 was defined as a separate risk period. We also used pre-exposure periods comprising −30 to −4 and −3 to −1 days relative to COVID-19 date to explore possible reverse causation in case the likelihood of the exposure (COVID-19) was increased by the occurrence of the outcome (arrhythmia), or in case there was delay of testing. The remaining time during the study period comprised the control (baseline) period (*Figure 1*). The death adjusted SCCS method^11^ was used. Individuals who died during the study period were included in the analyses and were censored on the date of death.

Moreover, we performed stratification analyses based on sex; age (>60 years and ≤60 years); pandemic wave; vaccination status; and COVID-19 disease severity. Pandemic waves were defined as follows: first wave 1 February to 31 July 2020; second wave 1 August 2020 to 31 January 2021; and third wave 1 February to 25 May 2021. Vaccinated individuals were defined as individuals who had received at least one dose of SARS-COV-2 vaccination at least 2 weeks before the COVID-19 date. Stratification analyses based on COVID-19 disease severity were performed. The individuals were stratified according to severity of disease, however, there were some categories with too few events, therefore the disease stratification differed. For PSVT and BA, disease severity was defined as hospitalized and non-hospitalized individuals. For AT, sample size allowed for stratification based on a four-scale disease severity as follows: no hospitalization; hospitalization; non-invasive ventilation and high-flow oxygen; and intensive care.

The analyses were performed using the package ‘SCCS’ version 1.4^13^ in R version 4.0.2. Sample size calculations were performed *a priori*. With 90% power, the sample size needed to identify a clinically relevant acute effect (IRR = 2), within a risk period of 30 days, was 181 events. The sample size needed to identify a clinically significant effect (IRR of 1.5) within a risk period of 180 days was 258 events. Due to data security issues, to ensure no individuals can be identified, only results with at least three or more individuals are shown. Therefore, subgrouping of variables if <3 individuals was performed after analysis.

### Matched cohort study

In the MCS,^14^ unadjusted and adjusted conditional Poisson regression analyses were performed to calculate the risk ratio (RR) of a first arrhythmia event in the risk period 1–30 days following COVID-19 date. To avoid bias due to ‘Day 0’, we did not include Day 0 in the study period. In contrast to the SCCS, all individuals are included in the analyses, both regarding the exposure (exposed and unexposed) and the outcome (individuals with and without an outcome event during the study period). We excluded individuals who died before the index date and individuals who died during the study period but had no arrhythmia event.

Potential confounders included (see supplementary material online, table  *S2*) as follows: heart failure and cardiomyopathies; acute and chronic ischaemic heart disease, obstructive sleep apnoea, and hyperthyroidism; treatment with anti-arrhythmics (defined by at least two filled prescriptions within 12 months prior to COVID-19/index date of pharmaceuticals with anatomical therapeutic chemical codes C07A and C01B in the National Prescribed Drug Register); vaccination status (defined as individuals who had received at least one dose of SARS-COV-2 vaccination at least two weeks before the COVID-19 date or index date); and comorbidities based on the weighted Charlson comorbidity index (wCCI).^15^ The wCCI was calculated for each individual based on data from the registers, using a method specifically adapted to Swedish national registers.^16^

Subgroup analyses were performed based on pandemic wave (first, second, and third waves) and COVID-19 disease severity (hospitalized and non-hospitalized individuals).The analyses were performed using the *g*, package gnm version 1.1–1^17^ in R, version 4.0.2 and IBM SPSS statistics software version 28. Sample size calculations were performed *a priori*. With 80% power, the sample size needed to identify a clinically relevant effect (RR = 2), within a risk period of 30 days, was 83 events.

## Results

**Figure 2:**
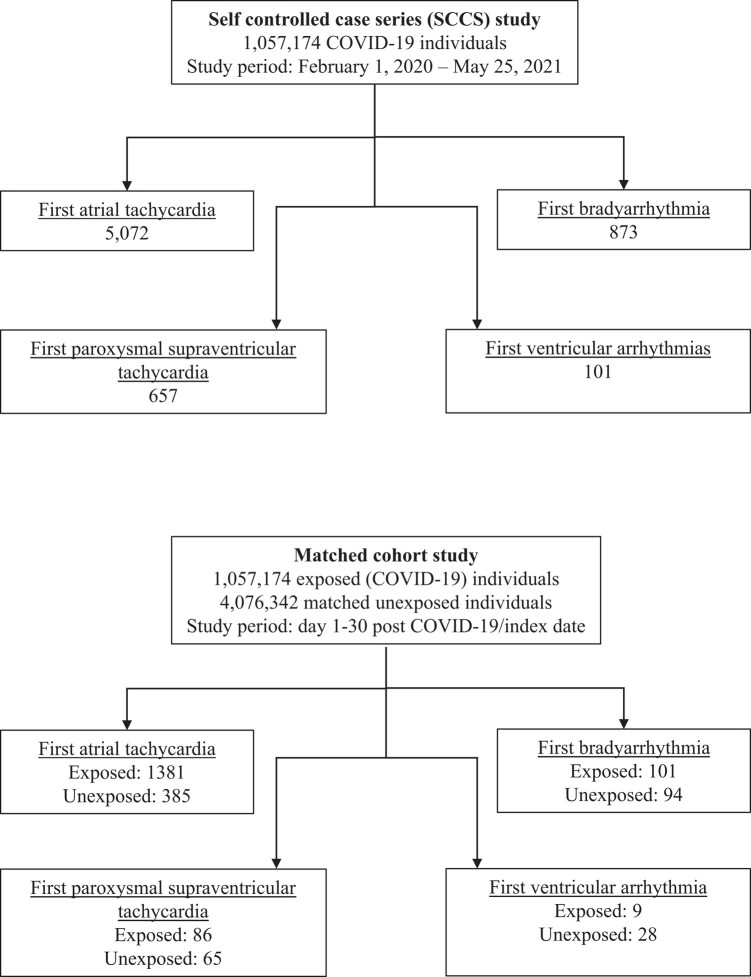
Overview of the self-controlled case series (SCCS) study design, including the whole study period, risk periods, pre-exposure periods, and control periods.

We analysed data on 1 057 174 individuals with COVID-19, as well as 4 076 342 matched unexposed individuals (*Figure 2*). Regarding the exposed (COVID-19) cohort, the median age was 39 [interquartile range (IQR) 25–53] years; 517 434 (49%) were males, and 539 740 (51%) were females; the majority (95%) did not need hospitalization (*Table 1*); and 19 073 (1.8%) died during the study period (*Table 1*).

**Table 1:** Descriptive characteristics of the study populations for the different cardiac arrhythmia outcomes in the self-controlled case series studies and matched cohort studies

			Atrial tachycardia			Paroxysmal supraventricular tachycardia			Bradyarrhythmias			Ventricular arrhythmias		
	Entire study population		MCS		SCCS	MCS		SCCS	MCS		SCCS	MCS		SCCS
Variable	Unexposed, *N* = 4 076 342	Exposed, *N* = 1 057 174	Unexposed, *N* = 3 875 326	Exposed, *N* = 1 021 424	*N* = 5072	Unexposed, *N* = 3 972 401	Exposed, *N* = 1 036 572	*N* = 657	Unexposed, *N* = 3 987 245	Exposed, *N* = 1 038 589	*N* = 873	Unexposed, *N* = 4 014 350	Exposed, *N* = 1 042 411	*N* = 101
Events, *n* (%)														
No	4 072 846 (99.914)	1 049 733 (99.296)	3 874 941 (99.990)	1 020 043 (99.865)		3 972 336 (99.998)	1 036 486 (99.992)		3 987 151 (99.998)	1 038 488 (99.990)		4 014 322 (99.999)	1 042 402 (99.999)	
Yes	3496 (0.086)	7441 (0.704)	385 (0.010)	1381 (0.135)		65 (0.002)	86 (0.008)		94 (0.002)	101 (0.010)		28 (0.001)	9 (0.001)	
Age in years, median (IQR)	39.00 (25.00–53.00)	39.00 (25.00–53.00)	38.00 (25.00–51.00)	38.00 (25.00–52.00)	75.00 (64.00–84.00)	39.00 (25.00–53.00)	39.00 (25.00–52.00)	53.00 (36.00–65.00)	39.00 (25.00–52.00)	39.00 (25.00–52.00)	77.00 (65.00–85.00)	39.00 (25.00–53.00)	39.00 (25.00–53.00)	60.00 (49.00–73.00)
Sex, *n* (%)														
Women	2 075 369 (50.913)	539 740 (51.055)	1 992 518 (51.415)	524 600 (51.360)	2080 (41.009)	2 023 286 (50.934)	529 722 (51.103)	356 (54.186)	2 034 999 (51.038)	531 310 (51.157)	315 (36.082)	2 047 541 (51.006)	533 084 (51.140)	29 (28.713)
Men	2 000 973 (49.087)	517 434 (48.945)	1 882 808 (48.585)	496 824 (48.640)	2992 (58.991)	1 949 115 (49.066)	506 850 (48.897)	301 (45.814)	1 952 246 (48.962)	507 279 (48.843)	558 (63.918)	1 966 809 (48.994)	509 327 (48.860)	72 (71.287)
Deaths, *n* (%)														
No	4 062 667 (99.665)	1 038 101 (98.196)	3 870 661 (99.880)	1 017 647 (99.630)	3931 (77.504)	3 964 405 (99.799)	1 031 516 (99.512)	623 (94.825)	3 980 007 (99.818)	1 033 817 (99.541)	693 (79.381)	4 006 161 (99.796)	1 037 290 (99.509)	86 (85.149)
Yes	13 675 (0.335)	19 073 (1.804)	4665 (0.120)	3777 (0.370)	1141 (22.496)	7996 (0.201)	5056 (0.488)	34 (5.175)	7238 (0.182)	4772 (0.459)	180 (20.619)	8189 (0.204)	5121 (0.491)	15 (14.851)
Severity, *n* (%)														
Unexposed	4 076 342	—	3 875 326	—		3 972 401	—		3 987 245	—		4 014 350	—	
Non-hospitalized	—	999 113 (94.508)	—	977 501 (95.700)	2214 (43.651)	—	987 296 (95.246)	508 (77.321)	—	989 980 (95.320)	438 (50.172)	—	992 612 (95.223)	68 (67.327)
Hospitalized	—	44 192 (4.180)	—	34 014 (3.330)	1952 (38.486)	—	38 638 (3.727)	109 (16.591)	—	38 020 (3.661)	335 (38.373)	—	39 077 (3.749)	18 (17.822)
Non-invasive ventilation/high-flow oxygen	—	6087 (0.576)	—	4098 (0.401)	363 (7.157)	—	4485 (0.433)	15 (2.283)	—	4435 (0.427)	46 (5.269)	—	4512 (0.433)	3 (2.970)
Intensive care	—	7782 (0.736)	—	5811 (0.569)	543 (10.706)	—	6153 (0.594)	25 (3.805)	—	6154 (0.593)	54 (6.186)	—	6210 (0.596)	12 (11.881)
Vaccination														
No, *n* (%)	3 959 178 (97.126)	1 042 055 (98.570)	3 769 860 (97.279)	1 008 018 (98.688)	4849 (95.603)	3 859 838 (97.166)	1 022 140 (98.608)	640 (97.412)	3 874 744 (97.178)	1 024 247 (98.619)	828 (94.845)	3 899 961 (97.150)	1 027 814 (98.600)	99 (98.020)
Yes, *n* (%)	117 164 (2.874)	15 119 (1.430)	105 466 (2.721)	13 406 (1.312)	223 (4.397)	112 563 (2.834)	14 432 (1.392)	17 (2.588)	112 501 (2.822)	14 342 (1.381)	45 (5.155)	114 389 (2.850)	14 597 (1.400)	2 (1.980)

## Increased risk of atrial tachycardias following COVID-19

### Self-controlled case series study

We identified 5072 COVID-19 individuals with a first AT event during the study period. The median age was 75 (IQR 64–84) years; 2080 (41%) were female, and 2992 (59%) were male (*Table 1*). The IRR of first AT was significantly increased up to 60 days following COVID-19 compared to the control period and was also increased during the pre-exposure periods ([−3, −1] and [−30, −4] days relatively to COVID-19 date (*Table 2*)). Specifically, the IRR was 12.38 (11.17 to 13.72) and 9.5 (8.46 to 10.67) during the first and second weeks following COVID-19, respectively.

**Table 2:** Incidence rate ratio with 95% confidence intervals of a first arrhythmia event following COVID-19 in the self-controlled case series study, presented by pre-defined time periods in days relative to COVID-19 date

Period (days)	Atrial tachycardias			PSVT			Bradyarrhythmias		
	Events (No)	IRR (95% CI)	*P*-value	Events (No)	IRR (95% CI)	*P*-value	Events (No)	IRR (95% CI)	*P*-value
Control period	2026	1 (ref.)	—	373	1 (ref.)	—	474	1 (ref.)	—
−30 to −4	512	2.48 (2.22–2.75)	<0.001	41	1.4 (0.99.1.99)	0.058	104	2.29 (1.82–2.89)	<0.001
−3 to −1	34	3.8 (2.7–5.35)	<0.001	6	5.29 (2.33–12.02)	<0.001	8	4.44 (2.19–9.02)	<0.001
0	451	49.64 (44.24–55.7)	<0.001	20	17.12 (10.63–27.59)	<0.001	35	19.11 (13.3–27.45)	<0.001
1 to 7	650	12.38 (11.17–13.72)	<0.001	16	2.42 (1.43–4.08)	0.001	43	4.07 (2.92–5.69)	<0.001
8 to 14	441	9.5 (8.46–10.67)	<0.001	25	4.01 (2.59–6.19)	<0.001	30	3.19 (2.17–4.71)	<0.001
15 to 30	290	2.88 (2.52–3.3)	<0.001	45	3.25 (2.29–4.62)	<0.001	28	1.37 (0.92–2.05)	0.1202
31 to 60	230	1.42 (1.22–1.65)	<0.001	41	1.94 (1.34–2.8)	<0.001	49	1.39 (1.01–1.93)	0.0436
61 to 90	154	1.11 (0.93–1.33)	0.2503	27	1.61 (1.03–2.5)	0.0356	30	0.96 (0.64–1.43)	0.8335
91 to 180	284	0.91 (0.78–1.05)	0.1992	63	1.75 (1.21–2.51)	0.0026	72	1.12 (0.83–1.51)	0.4719
1 to 30	1381	6.51 (5.97–7.1)	<0.001	86	2.99 (2.25–3.96)	<0.001	101	2.33 (1.83–2.97)	<0.001

PSVT, paroxysmal supraventricular tachycardia.

Age, COVID-19 severity, vaccination status, and pandemic wave, but not sex, were significant effect modifiers. The IRRs for AT were increased in older individuals (see supplementary material online, table  *S3*); during the first and third pandemic waves compared to the second wave (see supplementary material online, table  *S4*); in unvaccinated individuals (see supplementary material online, table  *S5*); and in more severe COVID-19 cases (see supplementary material online, table  *S6*). More severe COVID-19 was associated not only with higher IRRs but also with a longer period after COVID-19 where the IRRs were significantly increased. During the first week following COVID-19, the risk of AT was 25-fold increased in hospitalized COVID-19 individuals; 30-fold increased in COVID-19 individuals requiring non-invasive ventilation or high-flow oxygen; and ∼100-fold increased in COVID-19 individuals treated in the ICU.

### Matched cohort study

**Figure 3:**
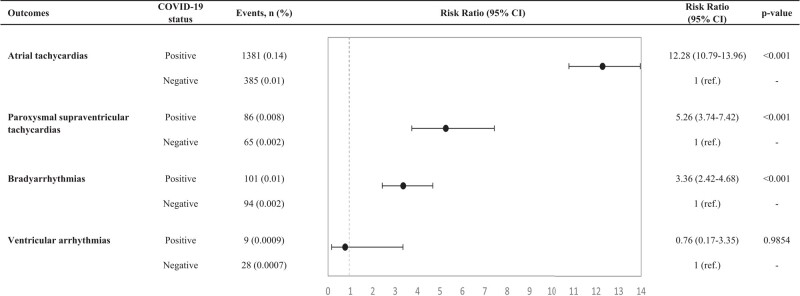
Adjusted relative risks with 95% confidence intervals of a first arrhythmia event within 30 days after COVID-19 in matched cohort study. Adjustment is performed for weighted Charlson comorbidity index score; heart failure and cardiomyopathies; ischaemic heart disease; hyperthyroidism; obstructive sleep apnoea; treatment with anti-arrhythmics; and vaccination. Total cohort consists of individuals who tested positive for SARS -CoV-2 and individuals without a positive PCR test for SARS-COV-2 (baseline group). Vertical dashed line indicates RR of 1.

We identified 1381 exposed and 385 unexposed individuals with a first AT event during the study period (*Table 1*). The AR of first AT during the study period was 0.135% for the exposed and 0.01% for the unexposed individuals, respectively (*Figure 3*). The RR of first AT for COVID-19 individuals compared to the background population was 12.28 (10.79–13.96) (see supplementary material online, table  *S7*). The RRs were higher during the first pandemic wave compared to the second and third waves (see supplementary material online, table  *S8*) and were further increased in patients with more severe COVID-19 (see supplementary material online, table  *S9*).

## Increased risk of paroxysmal supraventricular tachycardias following COVID-19

### Self-controlled case series study

During the study period, 657 COVID-19 individuals had a first PSVT event, where the median age was 53 (IQR 36–65) years; 356 (54%) were female, and 301 (46%) were male (*Table 1*). The IRR of first PSVT was significantly increased up to 180 days following COVID-19 with IRR of 2.42 (1.43 to 4.08) and 4.01 (2.50 to 6.19) during Days 1–7 and 8–14, respectively (*Table 2*). Age and COVID-19 severity had a significant effect on the association between COVID-19 and PSVT, while sex, vaccination status, and pandemic wave were not significant effect modifiers. The IRRs for PSVT were increased in older individuals (see supplementary material online, table  *S10*) and in severe COVID-19 cases (see supplementary material online, table  *S11*).

### Matched cohort study

We identified 86 COVID-19 individuals and 65 unexposed individuals with a first PSVT event during the study period (Days 1–30 following COVID-19) (*Table 1*). The AR of first AT during the study period was 0.008% for the COVID-19 and 0.002% for the unexposed individuals (*Figure 3*). The RR of first PSVT during the study period was 5.26 (3.74–7.42) (see supplementary material online, table  *S12*). The RRs increased depending on pandemic wave (see supplementary material online, table  *S13*) and COVID-19 severity (see supplementary material online, table  *S14*).

## Increased risk of bradyarrhythmias in COVID-19

### Self-controlled case series study

There were 873 COVID-19 individuals with a first BA event during the study period. The median age was 77 (IQR 65–85) years; 315 (36%) were female, and 558 (64%) were male (*Table 1*). The IRR of first BA was significantly increased up to two weeks following COVID-19 (*Table 2*). During the two first weeks after COVID-19, the IRR was 4.07 (2.92 to 5.69) and 3.19 (2.17 to 4.71), respectively. Significant effect modifiers were sex (see supplementary material online, table  *S15*); age (see supplementary material online, table  *S16*); vaccination status and pandemic wave (see supplementary material online, table  *S17*); and COVID-19 severity (see supplementary material online, table  *S18*).

### Matched cohort study

We identified 101 COVID-19 and 94 unexposed individuals with a first BA event during the study period (*Table 1*). The AR of first BA during the study period was 0.01% for COVID-19 and 0.002% for unexposed individuals (*Figure 3*). The adjusted RR of first BA during the study period was 3.36 (2.42–4.68) (see supplementary material online, table  *S19*); was higher during the first pandemic wave compared to the second and third waves (see supplementary material online, table  *S20*); and further increased in severe COVID-19 patients (see supplementary material online, table  *S21*). Interestingly, the RRs were not significantly increased in non-hospitalized individuals [0.8 (0.44–1.43)].

## Risk of ventricular arrhythmias following COVID-19

The risk of VA after COVID-19 was not found to be increased either in the SCCS study (see supplementary material online, table  *S22*) or the MCS (see supplementary material online, table  *S23*), apart from an increase during Day 0 (i.e. the COVID-19 date).

## Discussion

In this total-population study incorporating all laboratory-verified SARS-CoV-2 test positive individuals, we showed that COVID-19 increases the risk of first ever cardiac arrhythmias, apart from VAs.

We observed a high incidence of arrhythmia events whose date of occurrence coincides with the COVID-19 date (Day 0). We have previously reported this ‘day zero-effect’^5^ and have shown that bias was introduced by including ‘day-zero events’ in the risk periods following COVID-19.^12^ Thus, we excluded Day 0 from the risk periods following COVID-19 in both studies and used Day 0 as a separate period in the SCCS study, where the IRRs were the highest for all arrhythmia types. In addition, the risks were also increased during the pre-exposure periods (days [−30, −4] and [−3, −1]), possibly due to delayed testing or documentation of COVID-19 diagnosis or reverse causation, i.e. nosocomial COVID-19 during hospitalization for an arrhythmia event.

Only the risk of VA was not found to be increased during the post-exposure risk periods. Whether there is an underlying pathophysiological explanation for this finding or if it is explained by methodological limitations is unclear. The sample size for this outcome was insufficient, due to the exclusion from the analyses of day-zero events and individuals with pre-existing cardiovascular diagnosis of the same form. In addition, the coverage of this outcome by the National Patient Register is incomplete, in case of out-of-hospital cardiac arrest. We chose to not include sudden cardiac death to the analyses as a high proportion of cases are due to other causes than VAs, but this may also have affected the results on VAs.

Patients with severe COVID-19 had not only considerably higher risks of arrhythmias but also a longer period after COVID-19 where the risks were increased. These findings are in line with previous studies on the risk of cardiovascular complications after COVID-19.^4,6^ It is also known that the risk of arrhythmias is increased even in non-COVID-19 ICU patients.^8^ Vaccination against COVID-19 was protective against AT and BA after COVID-19, probably due to protection against severe COVID-19,^18^ but no effect was observed on the other arrhythmias. Indeed, few events were observed in the vaccinated groups, as until the end of the study period, only 2% of the study population had received at least one vaccination dose. Age was a significant effect modifier of the association between COVID-19 and outcomes. This is not an unexpected finding, considering previous studies showing that advancing age is a risk factor both for arrhythmias^19^ and for severe COVID-19,^20^ which in turn increases the risk of arrhythmic complications. Lastly, we compared the risk of outcomes among the different pandemic waves, but no specific pattern was identified. The risks were slightly higher in different pandemic waves depending on arrhythmia type and the study methodology.

Our findings of a temporal increased risk of cardiac arrhythmias following COVID-19 are corroborated by studies of other infectious diseases such as influenza and sepsis.^21–24^ In our study, the risk of arrhythmias was not only higher but also stayed increased for a longer period after the infection, which may be related to the underlying pathophysiology of COVID-19. There are several factors that occur during COVID-19 that could provide a plausible explanation for the association between COVID-19 and cardiac arrhythmias. Arrhythmias, for example PSVT, have a specific pathophysiology, mainly underlying anatomical disturbances of the electrical conduction system of the heart, that could not have been caused by the infection, which rather acted as trigger for arrhythmia events. These mechanisms include direct and indirect effects of the virus on the myocardium as well as extracardiac conditions that can trigger arrhythmogenesis in susceptible individuals.^7^ Specifically, COVID-19 may cause direct myocardial injury caused by viral invasion to the myocardial cells,^25^ or immune cell-mediated injury.^7,26^ However, COVID-19-related myocarditis is uncommon.^27^ Moreover, indirect myocardial injury may be caused by respiratory failure and hypoxia^28^ or systemic inflammatory response syndrome,^29^ common conditions in severe COVID-19. In addition, cardiovascular complications, such as myocardial infarction, ischaemic stroke,^5^ venous thromboembolism,^6^ or heart failure,^4^ are not uncommon in COVID-19 patients and could predispose these individuals to the development of cardiac arrhythmias, or could even be a complication of arrhythmic events. Metabolic disturbances during COVID-19, such as electrolyte derangements and intravascular volume imbalances, that have been observed during severe COVID-19, may also lead to arrhythmogenesis.^7^ Lastly, iatrogenic arrhythmias can be induced by specific COVID-19 medications (e.g. hydroxychloroquine), which prolong the QT interval,^30^ or by treatment with inotropes and vasopressors in severe COVID-19.^31^

Our findings that COVID-19 is a risk factor for cardiac arrhythmias are in line with several other studies.^4,9,10,32–34^ None of these, however, included all laboratory-verified SARS-CoV-2 positive test individuals in a country. Including all laboratory-verified SARS-CoV-2 test positive individuals avoids selection bias at entry to the study. Previous evidence on the epidemiology of COVID-19-related arrhythmias has been mainly performed in single-centre studies including exclusively hospitalized patients.^9,34^ In meta-analyses, the reported pooled prevalence was 5% both for atrial fibrillation^9,10^ and VAs,^34^ compared to the ARs in our study, which were much lower (0.135% and 0.001%, respectively).To improve statistical stringency, we included only the first event recorded during our study period and we only investigate the time period Days 1–30 following index date, which could explain the differences observed. We included the entire population in Sweden, thereby including individuals with asymptomatic/mild COVID-19. The denominator for the AR in our study is therefore larger than for other single-/multi-centre studies that may only include hospitalized patients. A large SCCS study from England investigated the risk of arrhythmias within 1–28 days following a SARS-CoV-2 positive test.^32^ In this period, the risk was 5 times increased for AT and for BA; 3 times increased for ventricular tachycardias and 3.5 for ventricular fibrillation, while PSVT was not studied separately. The reported risks were comparable to ours regarding AT (IRR 6.5 in our study), but slightly higher for BA (IRR 2.3 in our study) and significantly higher for VA, where the risks were not increased at all in our study. Nevertheless, in the English study, only vaccinated individuals who were hospitalized or died due to an arrhythmia were included, while in our study, we included all individuals with an arrhythmia, irrespectively of vaccination status or hospitalization due to arrhythmia. Two large cohort studies estimated the long-term cardiovascular outcomes in COVID-19 survivors.^4,33^ These studies are not directly comparable with our study due to different risk periods (months 1–12 after COVID-19), but they also show that COVID-19 (and especially severe disease) is associated with increased risk of arrhythmias. Despite large study populations, these studies were not total-population studies; indeed, one of them included mainly white males.^4^

We should also acknowledge limitations to our study. This study cannot prove causality due to its observational nature. Moreover, there is always a risk for incomplete or inaccurate data, misclassification bias, and residual confounding in register-based studies. However, if any type of misclassification bias existed, it would likely lead to underestimation of the associations (e.g. in case of differential misclassification of the exposure) or bias towards the null (e.g. in case of non-differential misclassification of the outcome), but likely not overestimation of the risks. Residual confounding may exist, for example we did not have information about smoking habits or diagnoses mainly managed in the primary health care, such as hypertension. This could also explain the slightly different relative risks between the SCCS and MCS. Unmeasured confounding could, indeed, exist for the MCS, but not for the SCCS, which automatically controls even for unmeasured time-fixed confounders. Nevertheless, the findings remain robust, due to the magnitude of the effects found, and the same direction of the associations, leading to similar conclusions.

With post-exposure risk period up to 180 days following the infection, the long-term risks of arrhythmias following the infection could not be explored. Future studies are, therefore, needed to investigate the risk of arrhythmias after COVID-19 in the long-term. Lastly, due to insufficient sample size, the association between COVID-19 and VAs needs to be evaluated further by future studies. There is a risk for undiagnosed out-of-hospital cardiac arrest events, not covered by the National Patient Register.^35^ Future studies, including data on COVID-19 after the emergence of new variants of SARS-CoV-2 and with higher vaccination coverage in the population, are needed to investigate the effect of new variants or vaccination.

This study has several strengths. It is one of the largest studies and the only one incorporating all laboratory-verified SARS-CoV-2 test positive individuals in a country to determine the association between COVID-19 and arrhythmias. Thus, the risk of inclusion and selection bias is minimal, and we had a large sample size to provide sufficient statistical power for the analyses (except for VA). We calculated the risks, not only of the most prevalent arrhythmia (i.e. atrial fibrillation) but also of other clinically relevant arrhythmias, such as PSVT. Moreover, we used two different study methodologies to reduce the risk of bias and residual confounding. The SCCS study design in which time-fixed (measured or unmeasured) confounders, such as sex and chronic conditions, are well controlled for, as cases act as their own controls. The MCS allows for comparison to the background population, and the results were adjusted for the effect of important confounders. Both study methodologies have their strengths, and limitations, but they complement each other. The results were similar in both, thus, the validity of the associations found are strengthened.

This study has several implications. Vaccination or booster doses against (severe) COVID-19 should be recommended to prevent severe COVID-19 and, therefore, arrhythmias. Screening for arrhythmias after COVID-19 may be indicated, especially for high-risk individuals, such as those with severe COVID-19 or older individuals. Lastly, prompt management of arrhythmic complications should be initiated, such as anticoagulation in patients with atrial fibrillation or pacemaker implantation in patients with bradyarrhythmias. Such timely interventions are expected to improve prognosis and quality of life in patients with arrhythmias following COVID-19.

## Conclusions

This study shows that COVID-19 is an independent risk factor for arrhythmias, especially atrial fibrillation and flutter. Individuals with more severe COVID-19, unvaccinated individuals, and elderly, more vulnerable individuals are at higher risk. These findings are in accordance with previous evidence and indicate the importance of preventive and therapeutic strategies, such as vaccination to prevent severe COVID-19 or early review after COVID-19 to detect arrhythmias in high-risk individuals.

## Supplementary Material

oead120_Supplementary_Data

## Data Availability

The data underlying this article will be shared on reasonable request to the corresponding author. The study used secondary registry data, which is regulated by the Public Access to Information and Secrecy Act (2009:400) and is protected by strict confidentiality. Synthetic (i.e. depersonalized and jittered) data can be provided on request from the corresponding author. The study protocol (R script) for the SCCS and matched cohort study are available on request from the corresponding author.
