# Supplementary material for: Risk of arrhythmias following COVID-19: nationwide self-controlled case series and matched cohort study
Source: Eur Heart J Open. 2023 Nov 21;3(6):oead120. doi: 10.1093/ehjopen/oead120 (PMC10711544; doi:10.1093/ehjopen/oead120)
Supplement: oead120_Supplementary_Data [file oead120_supplementary_data.zip › Suplemental Publication Material (Tables).docx]

Supplementary table 1: International Classification of Diseases (ICD) diagnosis and intervention codes used to identify the first event of cardiac arrhythmia.

| **Outcome** | **ICD9**  **(1987-1996)** | **ICD10**  **(1997 – to date)** | **Comment** |
| --- | --- | --- | --- |
| Atrial tachycardias | 4273 | I480  I481  I482  I483  I484  I489 | Diagnosis codes |
| Paroxysmal supraventricular tachycardias | 4267  4270 | I471  I456 | Diagnosis codes |
| Ventricular arrythmias | 4271  4274 | I470  I472A  I490 | Diagnosis codes |
| Brady-arrhythmias | 4260  4261.2  4261.3  4278 | I495  I441  I442 | Diagnosis codes |
|  | - | FPE00  FPE10  FPE20 | Intervention codes |

Supplementary table 2: International Classification of Diseases (ICD-10) diagnosis codes used to identify confounders in the matched cohort study.

| **Variable** | **ICD-10 codes** |
| --- | --- |
| Heart failure & cardiomyopathies | I420, I421, I422, I423, I424, I425, I426, I427, I428, I429, I430, I431, I432, I438, I500, I501, I509 |
| Ischemic heart disease (acute, chronic) | I200, I201, I208, I209, I210, I211, I212, I213, I214, I219, I220, I221, I228, I229, I240, I248, I249, I250, I251, I252, I253, I254, I255, I256, I258, I259 |
| Hyperthyroidism | E050, E051, E052, E053, E054, E055, E058, E059 |
| Obstructive sleep apnea | G473 |

Supplementary table 3: Effect modification for atrial tachycardias by age (<=60 years, and >60 years) in the self-controlled case series (SCCS) study, presented by predefined times periods in days relative to COVID-19 date. IRR: Incidence Rate Ratio; CI: Confidence Intervals.

|  | <=60 | | | >60 | | |
| --- | --- | --- | --- | --- | --- | --- |
| Period (days) | Events,  No | IRR (95%CI) | P value | Events,  No | IRR (95%CI) | P value |
| Control period | 496 | 1 (ref) |  | 1,530 | 1 (ref) |  |
| -30 to -4 | 56 | 1.49 (1.11 to 2) | 0.0072 | 456 | 2.95 (2.63 to 3.31) | <0.001 |
| -3 to -1 | 2 | 1.39 (0.35 to 5.59) | 0.6438 | 32 | 4.97 (3.48 to 7.08) | <0.001 |
| 0 | 53 | 36.89 (27.27 to 49.9) | <0.001 | 398 | 59.25 (52.31 to 67.1) | <0.001 |
| 1 to 7 | 75 | 8.79 (6.74 to 11.46) | <0.001 | 575 | 13.83 (12.35 to 15.48) | <0.001 |
| 8 to 14 | 70 | 8.33 (6.33 to 10.95) | <0.001 | 371 | 9.84 (8.66 to 11.2) | <0.001 |
| 15 to 30 | 56 | 2.81 (2.08 to 3.8) | <0.001 | 234 | 2.94 (2.53 to 3.42) | <0.001 |
| 31 to 60 | 54 | 1.64 (1.2 to 2.24) | 0.0017 | 176 | 1.43 (1.21 to 1.7) | <0.001 |
| 61 to 90 | 34 | 1.22 (0.84 to 1.78) | 0.3033 | 120 | 1.15 (0.94 to 1.41) | 0.1688 |
| 91 to 180 | 84 | 1.25 (0.93 to 1.67) | 0.1389 | 200 | 0.87 (0.73 to 1.03) | 0.1057 |

Supplementary table 4: Effect modification for atrial tachycardias by pandemic wave in the self-controlled case series study, presented by predefined times periods in days relative to COVID-19 date. Pandemic waves were defined as follows: first wave February 1 to July 31, 2020; second wave August 1, 2020, to January 31, 2021; and third wave February 1 to May 25, 2021. IRR: Incidence Rate Ratio; CI: Confidence Intervals.

|  | Wave 1 | | | Wave 2 | | | Wave 3 | | |
| --- | --- | --- | --- | --- | --- | --- | --- | --- | --- |
| Period (days) | Events, No | IRR  (95%CI) | P value | Events, No | IRR (95%CI) | P value | Events, No | IRR (95%CI) | P value |
| Control period | 265 | 1 (ref) |  | 1,028 | 1 (ref) |  | 733 | 1 (ref) |  |
| -30 to -4 | 125 | 2.47 (1.87 to 3.26) | <0.001 | 273 | 1.89 (1.58 to 2.28) | <0.001 | 114 | 1.86 (1.38 to 2.5) | <0.001 |
| -3 to -1 | 9 | 4.28 (2.14 to 8.59) | <0.001 | 15 | 2.34 (1.38 to 3.97) | 0.0017 | 10 | 4.18 (2.12 to 8.23) | <0.001 |
| 0 | 155 | 71.94 (53.82 to 96.15) | <0.001 | 193 | 29.22 (23.58 to 36.22) | <0.001 | 103 | 43.12 (30.69 to 60.6) | <0.001 |
| 1 to 7 | 168 | 11.79 (8.79 to 15.83) | <0.001 | 315 | 8.12 (6.66 to 9.91) | <0.001 | 167 | 11.97 (8.66 to 16.56) | <0.001 |
| 8 to 14 | 100 | 7.77 (5.59 to 10.79) | <0.001 | 213 | 6.14 (4.94 to 7.63) | <0.001 | 128 | 9.87 (6.99 to 13.94) | <0.001 |
| 15 to 30 | 62 | 2.44 (1.69 to 3.55) | <0.001 | 155 | 2.02 (1.59 to 2.57) | <0.001 | 73 | 2.59 (1.74 to 3.84) | <0.001 |
| 31 to 60 | 40 | 1.11 (0.72 to 1.7) | 0.6326 | 135 | 1.09 (0.83 to 1.41) | 0.5415 | 55 | 1.41 (0.9 to 2.21) | 0.1361 |
| 61 to 90 | 27 | 0.93 (0.56 to 1.53) | 0.7694 | 116 | 1.04 (0.77 to 1.39) | 0.8094 | 11 | 0.6 (0.29 to 1.24) | 0.1665 |
| 91 to 180 | 76 | 0.99 (0.67 to 1.47) | 0.9768 | 203 | 0.81 (0.6 to 1.09) | 0.1601 | 5 | 1.34 (0.47 to 3.8) | 0.5814 |

Supplementary table 5: Effect modification for atrial tachycardias by vaccination status in the self-controlled case series study, presented by predefined times periods in days relative to COVID-19 date. IRR: Incidence Rate Ratio; CI: Confidence Intervals.

|  | Unvaccinated | | | Vaccinated | | |
| --- | --- | --- | --- | --- | --- | --- |
| Period (days) | Events, No | IRR (95%CI) | P value | Events, No | IRR (95%CI) | P value |
| Control period | 1,883 | 1 (ref) |  | 143 | 1 (ref) |  |
| -30 to -4 | 495 | 2.54 (2.28 to 2.83) | <0.001 | 17 | 1.24 (0.62 to 2.47) | 0.5426 |
| -3 to -1 | 31 | 3.68 (2.57 to 5.27) | <0.001 | 3 | 5.3 (1.5 to 18.69) | 0.0096 |
| 0 | 432 | 50.54 (44.92 to 56.86) | <0.001 | 19 | 33.31 (16.2 to 68.48) | <0.001 |
| 1 to 7 | 627 | 12.67 (11.41 to 14.08) | <0.001 | 23 | 7.15 (3.51 to 14.57) | <0.001 |
| 8 to 14 | 436 | 9.9 (8.81 to 11.14) | <0.001 | 5 | 1.85 (0.63 to 5.37) | 0.2615 |
| 15 to 30 | 283 | 2.94 (2.57 to 3.37) | <0.001 | 7 | 1.34 (0.5 to 3.6) | 0.5649 |
| 31 to 60 | 226 | 1.44 (1.24 to 1.67) | <0.001 | 6* | 0.58 (0.19 to 1.79) | 0.3410 |
| 61 to 90 | 153 | 1.12 (0.94 to 1.34) | 0.2040 |  |  |  |
| 91 to 180 | 283 | 0.91 (0.78 to 1.05) | 0.2048 |  |  |  |

*risk period from 31 to 180 days

Supplementary table 6: Effect modification for atrial tachycardias by COVID-19 severity in the self-controlled case series study, presented by predefined times periods in days relative to COVID-19 date. COVID-19 severity defined as: no hospitalization; hospitalization; non-invasive ventilation and high-flow oxygen; intensive care. IRR: Incidence Rate Ratio; CI: Confidence Intervals.

|  | Non-hospitalized | | | Hospitalized | | | Non-invasive ventilation & high-flow oxygen | | | Intensive care | | |
| --- | --- | --- | --- | --- | --- | --- | --- | --- | --- | --- | --- | --- |
| Period (days) | Events, No | IRR (95%CI) | P value | Events, No | IRR (95%CI) | P value | Events, No | IRR (95%CI) | P value | Events, No | IRR (95%CI) | P value |
| Control period | 1,365 | 1 (ref) |  | 531 | 1 (ref) |  | 69 | 1 (ref) |  | 61 | 1 (ref) |  |
| -30 to -4 | 174 | 1.26 (1.06 to 1.5) | 0.0078 | 257 | 4.61 (3.92 to 5.43) | <0.001 | 46 | 5.53 (3.54 to 8.63) | <0.001 | 35 | 5.68 (3.6 to 8.99) | <0.001 |
| -3 to -1 | 10 | 1.84 (0.98 to 3.45) | 0.0564 | 20 | 8.27 (5.25 to 13.01) | <0.001 | 3 | 6.93 (2.11 to 22.74) | 0.0014 | 1 | 3.56 (0.49 to 26.03) | 0.2105 |
| 0 | 42 | 7.68 (5.59 to 10.54) | <0.001 | 288 | 116.26 (98.69 to 136.97) | <0.001 | 50 | 111.48 (70.43 to 176.46) | <0.001 | 71 | 259.86 (172.84 to 390.71) | <0.001 |
| 1 to 7 | 44 | 1.4 (1.03 to 1.91) | 0.0324 | 357 | 24.92 (21.33 to 29.11) | <0.001 | 90 | 32.91 (21.57 to 50.19) | <0.001 | 159 | 97.36 (67.17 to 141.1) | <0.001 |
| 8 to 14 | 44 | 1.51 (1.11 to 2.07) | 0.0090 | 224 | 17.87 (15 to 21.29) | <0.001 | 61 | 25.94 (16.55 to 40.67) | <0.001 | 112 | 69.75 (47.23 to 103.01) | <0.001 |
| 15 to 30 | 104 | 1.55 (1.24 to 1.92) | <0.001 | 107 | 3.8 (3.04 to 4.75) | <0.001 | 21 | 4.64 (2.62 to 8.23) | <0.001 | 58 | 16.24 (10.48 to 25.16) | <0.001 |
| 31 to 60 | 138 | 1.18 (0.97 to 1.44) | 0.1048 | 54 | 1.11 (0.83 to 1.5) | 0.4733 | 12 | 1.68 (0.84 to 3.36) | 0.1397 | 26 | 5.42 (3.17 to 9.26) | <0.001 |
| 61 to 90 | 93 | 0.9 (0.71 to 1.14) | 0.3966 | 44 | 1.03 (0.75 to 1.43) | 0.8435 | 6 | 0.92 (0.37 to 2.29) | 0.8537 | 11 | 2.99 (1.46 to 6.12) | 0.0028 |
| 91 to 180 | 202 | 0.9 (0.74 to 1.1) | 0.2946 | 67 | 0.69 (0.52 to 0.91) | 0.0100 | 5 | 0.32 (0.12 to 0.89) | 0.0296 | 10 | 1.09 (0.5 to 2.35) | 0.8322 |

Supplementary table 7: Unadjusted and adjusted conditional Poisson regression models for first atrial tachycardia event within 30 days after COVID-19 in matched cohort study.

| Variables | Levels | Atrial tachycardia, n (%) | Univariable RR  (95%CI) | p-value | Multivariable RR (95%CI) | p-value |
| --- | --- | --- | --- | --- | --- | --- |
| COVID-19 | Neg. | 385 (0.0099) | 1 (ref) | - | 1 (ref) | - |
|  | Pos. | 1381 (0.1352) | 11.93 (10.65-13.37) | <0.001 | 12.28 (10.79-13.96) | <0.001 |
| wCCI | 0 | 1459 (0.0319) | 1 (ref) | - | 1 (ref) | - |
|  | 1-2 | 167 (0.0814) | 1.88 (1.54-2.3) | <0.001 | 1.46 (1.07-1.98) | 0.0163 |
|  | 3-4 | 57 (0.1792) | 3.66 (2.48-5.42) | <0.001 | 0.78 (0.43-1.43) | 0.4220 |
|  | >=5 | 83 (0.0928) | 1.95 (1.47-2.58) | <0.001 | 1.18 (0.75-1.86) | 0.4826 |
| Heart failure & cardiomyopathies | No | 1315 (0.0270) | 1 (ref) | - | 1 (ref) | - |
|  | Yes | 451 (1.4980) | 8.51 (7.05-10.28) | <0.001 | 8.88 (6.77-11.64) | <0.001 |
| Ischemic heart disease | No | 1385 (0.0288) | 1 (ref) | - | 1 (ref) | - |
|  | Yes | 381 (0.4769) | 1.88 (1.63-2.17) | <0.001 | 1 (0.78-1.27) | 0.9702 |
| Hyperthyroidism | No | 1725 (0.0355) | 1 (ref) | - | 1 (ref) | - |
|  | Yes | 41 (0.1101) | 1.85 (1.25-2.74) | 0.0021 | 1.77 (0.96-3.27) | 0.0667 |
| Obstructive sleep apnea | No | 1623 (0.0338) | 1 (ref) | - | 1 (ref) | - |
|  | Yes | 143 (0.1618) | 2.1 (1.69-2.6) | <0.001 | 1.38 (0.98-1.93) | 0.0636 |
| Anti-arrhythmics | No | 1114 (0.0237) | 1 (ref) | - | 1 (ref) | - |
|  | Yes | 652 (0.3278) | 2.2 (1.95-2.49) | <0.001 | 1.39 (1.14-1.7) | 0.0011 |
| Vaccination | No | 1715 (0.0359) | 1 (ref) | - | 1 (ref) | - |
|  | Yes | 51 (0.0429) | 0.5 (0.34-0.75) | <0.001 | 0.71 (0.4-1.25) | 0.2390 |

RR=Relative Risk. CI= Confidence Interval. wCCI=weighted Charlson Comorbidity Index.

Supplementary table 8: risk of first ever atrial tachycardia in the MCS stratified by pandemic wave.

| Wave | Variables | Levels | Atrial tachycardia, n (%) | Univariable RR (95%CI) | p-value | Multivariable RR (95%CI) | p-value |
| --- | --- | --- | --- | --- | --- | --- | --- |
| 1^st^ | COVID-19 | Neg. | 58 (0.0282) | 1 (ref) | - | 1 (ref) | - |
|  |  | Pos. | 319 (0.5316) | 17.48 (13.2-23.16) | <0.001 | 28.3 (16.21-49.41) | <0.001 |
|  | wCCI | 0 | 174 (0.0707) | 1 (ref) | - | 1 (ref) | - |
|  |  | 1-2 | 104 (0.9004) | 16.58 (10.25-26.82) | <0.001 | 0.76 (0.35-1.65) | 0.4913 |
|  |  | 3-4 | 43 (1.6190) | 13.37 (6.48-27.57) | <0.001 | 0.45 (0.16-1.22) | 0.1155 |
|  |  | >=5 | 56 (0.9913) | 14.27 (7.61-26.76) | <0.001 | 0.63 (0.25-1.6) | 0.3286 |
|  | Heart failure & cardiomyopathies | No | 258 (0.0986) | 1 (ref) | - | 1 (ref) | - |
|  |  | Yes | 119 (2.8239) | 10.18 (6.85-15.13) | <0.001 | 15.41 (7.93-29.95) | <0.001 |
|  | Ischemic heart disease | No | 288 (0.1122) | 1 (ref) | - | 1 (ref) | - |
|  |  | Yes | 89 (0.9696) | 2.13 (1.56-2.9) | <0.001 | 0.74 (0.39-1.39) | 0.3502 |
|  | Hyperthyroidism | No | 368 (0.1400) | 1 (ref) | - | 1 (ref) | - |
|  |  | Yes | 9 (0.3032) | 2.64 (1.08-6.45) | 0.0337 | 2.12 (0.46-9.78) | 0.3345 |
|  | Obstructive sleep apnea | No | 353 (0.1360) | 1 (ref) | - | 1 (ref) | - |
|  |  | Yes | 24 (0.3857) | 1.58 (0.96-2.61) | 0.0735 | 0.62 (0.23-1.69) | 0.3489 |
|  | Anti-arrhythmics | No | 233 (0.0946) | 1 (ref) | - | 1 (ref) | - |
|  |  | Yes | 144 (0.7348) | 2.16 (1.66-2.82) | <0.001 | 1.62 (0.96-2.72) | 0.0708 |
| 2^nd^ | COVID-19 | Neg. | 191 (0.0137) | 1 (ref) | - | 1 (ref) | - |
|  |  | Pos. | 545 (0.1424) | 9.32 (7.89-11) | <0.001 | 9.4 (7.81-11.31) | <0.001 |
|  | wCCI | 0 | 639 (0.0428) | 1 (ref) | - | 1 (ref) | - |
|  |  | 1-2 | 56 (0.0314) | 0.61 (0.45-0.83) | 0.0014 | 1.17 (0.79-1.73) | 0.4253 |
|  |  | 3-4 | 14 (0.0524) | 1.07 (0.57-1.99) | 0.8349 | 0.7 (0.28-1.77) | 0.4552 |
|  |  | >=5 | 27 (0.0351) | 0.65 (0.42-1.01) | 0.0530 | 1.09 (0.61-1.95) | 0.7686 |
|  | Heart failure & cardiomyopathies | No | 560 (0.0317) | 1 (ref) | - | 1 (ref) | - |
|  |  | Yes | 176 (1.4805) | 7.58 (5.65-10.17) | <0.001 | 6.37 (4.32-9.4) | <0.001 |
|  | Ischemic heart disease | No | 573 (0.0328) | 1 (ref) | - | 1 (ref) | - |
|  |  | Yes | 163 (0.5197) | 1.82 (1.46-2.26) | <0.001 | 1.23 (0.88-1.72) | 0.2185 |
|  | Hyperthyroidism | No | 723 (0.0410) | 1 (ref) | - | 1 (ref) | - |
|  |  | Yes | 13 (0.0909) | 1.31 (0.67-2.53) | 0.4291 | 1.54 (0.63-3.73) | 0.3412 |
|  | Obstructive sleep apnea | No | 680 (0.0390) | 1 (ref) | - | 1 (ref) | - |
|  |  | Yes | 56 (0.1715) | 1.93 (1.38-2.71) | <0.001 | 1.18 (0.72-1.93) | 0.5144 |
|  | Anti-arrhythmics | No | 472 (0.0278) | 1 (ref) | - | 1 (ref) | - |
|  |  | Yes | 264 (0.3446) | 2.08 (1.72-2.51) | <0.001 | 1.42 (1.07-1.88) | 0.0164 |
| 3^rd^ | COVID-19 | Neg. | 136 (0.0060) | 1 (ref) | - | 1 (ref) | - |
|  |  | Pos. | 517 (0.0893) | 13.24 (10.95-16) | <0.001 | 14.4 (11.55-17.94) | <0.001 |
|  | wCCI | 0 | 646 (0.0228) | 1 (ref) | - | 1 (ref) | - |
|  |  | 1-2 | 7 (0.0453) | 1.02 (0.4-2.59) | 0.9682 | 3.92 (1.05-14.58) | 0.0418 |
|  |  | 3-4 | 0 (0.0000) | 0 (0-inf.) | 0.9483 | 0 (0-inf.) | 0.9658 |
|  |  | >=5 | 0 (0.0000) | 0 (0-inf.) | 0.8873 | 0 (0-inf.) | 0.9570 |
|  | Heart failure & cardiomyopathies | No | 497 (0.0175) | 1 (ref) | - | 1 (ref) | - |
|  |  | Yes | 156 (1.1139) | 8.61 (6.29-11.79) | <0.001 | 11.09 (6.91-17.8) | <0.001 |
|  | Ischemic heart disease | No | 524 (0.0186) | 1 (ref) | - | 1 (ref) | - |
|  |  | Yes | 129 (0.3279) | 1.82 (1.44-2.31) | <0.001 | 0.85 (0.55-1.31) | 0.4545 |
|  | Hyperthyroidism | No | 634 (0.0224) | 1 (ref) | - | 1 (ref) | - |
|  |  | Yes | 19 (0.0952) | 2.15 (1.19-3.88) | 0.0111 | 1.99 (0.71-5.6) | 0.1938 |
|  | Obstructive sleep apnea | No | 590 (0.0210) | 1 (ref) | - | 1 (ref) | - |
|  |  | Yes | 63 (0.1273) | 2.62 (1.86-3.69) | <0.001 | 2.15 (1.25-3.68) | 0.0054 |
|  | Anti-arrhythmics | No | 409 (0.0149) | 1 (ref) | - | 1 (ref) | - |
|  |  | Yes | 244 (0.2376) | 2.38 (1.95-2.9) | <0.001 | 1.29 (0.92-1.82) | 0.1351 |
|  | Vaccination | No | 602 (0.0220) | 1 (ref) | - | 1 (ref) | - |
|  |  | Yes | 51 (0.0429) | 0.5 (0.34-0.75) | <0.001 | 0.73 (0.4-1.32) | 0.2920 |

Supplementary table 9: risk of first ever atrial tachycardia in the MCS stratified by disease severity (hospitalized and non-hospitalized individuals).

| COVID-19 severity | Variables | Levels | Atrial tachycardia, n (%) | Univariable RR (95%CI) | p-value | Multivariable RR (95%CI) | p-value |
| --- | --- | --- | --- | --- | --- | --- | --- |
| Non-hospitalised | COVID-19 | Neg. | 311 (0.0084) | 1 (ref) | - | 1 (ref) | - |
|  |  | Pos. | 192 (0.0196) | 2.15 (1.79-2.57) | <0.001 | 2.52 (2.07-3.07) | <0.001 |
|  | wCCI | 0 | 453 (0.0103) | 1 (ref) | - | 1 (ref) | - |
|  |  | 1-2 | 37 (0.0190) | 1.38 (0.93-2.06) | 0.1120 | 1.45 (0.94-2.25) | 0.0943 |
|  |  | 3-4 | 2 (0.0068) | 0.5 (0.11-2.23) | 0.3641 | 0.41 (0.09-1.9) | 0.2562 |
|  |  | >=5 | 11 (0.0130) | 0.59 (0.3-1.16) | 0.1242 | 0.69 (0.33-1.46) | 0.3314 |
|  | Heart failure & cardiomyopathies | No | 396 (0.0085) | 1 (ref) | - | 1 (ref) | - |
|  |  | Yes | 107 (0.4508) | 8.73 (6-12.71) | <0.001 | 9.48 (6.32-14.21) | <0.001 |
|  | Ischemic heart disease | No | 415 (0.0090) | 1 (ref) | - | 1 (ref) | - |
|  |  | Yes | 88 (0.1357) | 1.94 (1.45-2.59) | <0.001 | 1.11 (0.77-1.59) | 0.5842 |
|  | Hyperthyroidism | No | 492 (0.0106) | 1 (ref) | - | 1 (ref) | - |
|  |  | Yes | 11 (0.0315) | 1.94 (0.92-4.12) | 0.0831 | 2.19 (0.96-4.97) | 0.0621 |
|  | Obstructive sleep apnea | No | 467 (0.0101) | 1 (ref) | - | 1 (ref) | - |
|  |  | Yes | 36 (0.0453) | 1.92 (1.27-2.92) | 0.0021 | 1.64 (1.02-2.62) | 0.0394 |
|  | Anti-arrhythmics | No | 351 (0.0078) | 1 (ref) | - | 1 (ref) | - |
|  |  | Yes | 152 (0.0887) | 2.02 (1.6-2.57) | <0.001 | 1.39 (1.05-1.84) | 0.0228 |
|  | Vaccination | No | 489 (0.0107) | 1 (ref) | - | 1 (ref) | - |
|  |  | Yes | 14 (0.0124) | 0.75 (0.39-1.42) | 0.3766 | 0.67 (0.32-1.41) | 0.2951 |
| Hospitalised | COVID-19 | Neg. | 74 (0.0473) | 1 (ref) | - | 1 (ref) | - |
|  |  | Pos. | 1189 (2.7070) | 52.65 (41.61-66.61) | <0.001 | 55.09 (41.88-72.46) | <0.001 |
|  | wCCI | 0 | 1006 (0.5490) | 1 (ref) | - | 1 (ref) | - |
|  |  | 1-2 | 130 (1.2939) | 2.1 (1.66-2.66) | <0.001 | 0.88 (0.47-1.66) | 0.6972 |
|  |  | 3-4 | 55 (2.3656) | 4.92 (3.2-7.58) | <0.001 | 0.43 (0.16-1.11) | 0.0804 |
|  |  | >=5 | 72 (1.5148) | 2.92 (2.11-4.04) | <0.001 | 1.23 (0.48-3.15) | 0.6608 |
|  | Heart failure & cardiomyopathies | No | 919 (0.4737) | 1 (ref) | - | 1 (ref) | - |
|  |  | Yes | 344 (5.3978) | 8.44 (6.78-10.49) | <0.001 | 7.73 (4.5-13.29) | <0.001 |
|  | Ischemic heart disease | No | 970 (0.5234) | 1 (ref) | - | 1 (ref) | - |
|  |  | Yes | 293 (1.9471) | 1.86 (1.58-2.19) | <0.001 | 0.83 (0.51-1.35) | 0.4531 |
|  | Hyperthyroidism | No | 1233 (0.6225) | 1 (ref) | - | 1 (ref) | - |
|  |  | Yes | 30 (1.2993) | 1.82 (1.15-2.89) | 0.0107 | 1.21 (0.33-4.4) | 0.7692 |
|  | Obstructive sleep apnea | No | 1156 (0.6035) | 1 (ref) | - | 1 (ref) | - |
|  |  | Yes | 107 (1.2096) | 2.17 (1.68-2.79) | <0.001 | 1.75 (0.89-3.46) | 0.1062 |
|  | Anti-arrhythmics | No | 763 (0.4412) | 1 (ref) | - | 1 (ref) | - |
|  |  | Yes | 500 (1.8217) | 2.27 (1.97-2.62) | <0.001 | 1.32 (0.88-1.98) | 0.1778 |
|  | Vaccination | No | 1226 (0.6309) | 1 (ref) | - | 1 (ref) | - |
|  |  | Yes | 37 (0.6100) | 0.4 (0.24-0.67) | <0.001 | 1.09 (0.36-3.25) | 0.8821 |

Supplementary table 10: Effect modification for paroxysmal supraventricular tachycardias by age (<=60 years, and >60 years) in the self-controlled case series study, presented by predefined times periods in days relative to COVID-19 date. IRR: Incidence Rate Ratio; CI: Confidence Intervals.

|  | <=60 | | | >60 | | |
| --- | --- | --- | --- | --- | --- | --- |
| Period (days) | Events, No | IRR (95%CI) | P value | Events, No | IRR (95%CI) | P value |
| Control period | 260 | 1 (ref) |  | 113 | 1 (ref) |  |
| -30 to -4 | 25 | 1.29 (0.83 to 2) | 0.2532 | 16 | 1.61 (0.9 to 2.9) | 0.1102 |
| -3 to -1 | 3 | 4.13 (1.31 to 13.05) | 0.0157 | 3 | 7.57 (2.31 to 24.76) | <0.001 |
| 0 | 7 | 9.68 (4.48 to 20.91) | <0.001 | 13 | 31.93 (16.64 to 61.27) | <0.001 |
| 1 to 7 | 6 | 1.42 (0.62 to 3.25) | 0.4041 | 10 | 4.48 (2.18 to 9.19) | <0.001 |
| 8 to 14 | 9 | 2.19 (1.1 to 4.35) | 0.0258 | 16 | 8.08 (4.38 to 14.93) | <0.001 |
| 15 to 30 | 29 | 3.04 (1.98 to 4.66) | <0.001 | 16 | 3.89 (2.09 to 7.23) | <0.001 |
| 31 to 60 | 27 | 1.69 (1.08 to 2.64) | 0.0221 | 14 | 2.59 (1.34 to 5.01) | 0.0046 |
| 61 to 90 | 17 | 1.3 (0.75 to 2.24) | 0.3489 | 10 | 2.33 (1.08 to 4.99) | 0.0303 |
| 91 to 180 | 50 | 1.84 (1.21 to 2.79) | 0.0041 | 13 | 1.24 (0.59 to 2.6) | 0.5685 |

Supplementary table 11: Effect modification for paroxysmal supraventricular tachycardias by COVID-19 severity (non-hospitalised and hospitalised) in the self-controlled case series study, presented by predefined times periods in days relative to COVID-19 date. IRR: Incidence Rate Ratio; CI: Confidence Intervals.

|  | Not hospitalized | | | Hospitalized | | |
| --- | --- | --- | --- | --- | --- | --- |
| Period (days) | Events, No | IRR (95%CI) | P value | Events, No | IRR (95%CI) | P value |
| Control period | 322 | 1 (ref) |  | 51 | 1 (ref) |  |
| -30 to -4 | 36 | 1.47 (1.01 to 2.15) | 0.0462 | 6* | 1.18 (0.48 to 2.86) | 0.7204 |
| -3 to -1 | 5 | 5.49 (2.23 to 13.52) | 0.0002 |  |  |  |
| 0 | 5 | 5.49 (2.23 to 13.53) | 0.0002 | 15 | 69.61 (36.13 to 134.13) | <0.001 |
| 1 to 7 | 11** | 0.98 (0.52 to 1.84) | 0.9481 | 14 | 11.09 (5.67 to 21.66) | <0.001 |
| 8 to 14 |  |  |  | 16 | 13.45 (7.04 to 25.66) | <0.001 |
| 15 to 30 | 28 | 2.37 (1.54 to 3.66) | <0.001 | 17 | 6.82 (3.6 to 12.91) | <0.001 |
| 31 to 60 | 28 | 1.45 (0.93 to 2.25) | 0.1031 | 13 | 3.79 (1.88 to 7.61) | <0.001 |
| 61 to 90 | 18 | 1.14 (0.66 to 1.94) | 0.6433 | 9 | 3.23 (1.44 to 7.22) | 0.0044 |
| 91 to 180 | 55 | 1.68 (1.11 to 2.55) | 0.0140 | 8 | 1.01 (0.42 to 2.43) | 0.9768 |

* Buffer period from -30 to -1 days; ** risk period from 1 to 14 days

Supplementary table 12: Unadjusted and adjusted conditional Poisson regression models for first paroxysmal supraventricular tachycardia (PSVT) event within 30 days after COVID-19 in matched cohort study.

| Variables | Levels | PSVT, n (%) | Univariable RR  (95%CI) | p-value | Multivariable RR (95%CI) | p-value |
| --- | --- | --- | --- | --- | --- | --- |
| COVID-19 | Neg. | 65 (0.0016) | 1 (ref) | - | 1 (ref) | - |
|  | Pos. | 86 (0.0083) | 4.96 (3.59-6.85) | <0.001 | 5.26 (3.74-7.42) | <0.001 |
| wCCI | 0 | 137 (0.0029) | 1 (ref) | - | 1 (ref) | - |
|  | 1-2 | 10 (0.0047) | 1.2 (0.57-2.53) | 0.6315 | 0.81 (0.34-1.94) | 0.6400 |
|  | 3-4 | 1 (0.0030) | 0.91 (0.1-8.23) | 0.9343 | 0.45 (0.02-11.79) | 0.6350 |
|  | >=5 | 3 (0.0032) | 0.74 (0.21-2.63) | 0.6462 | 0.5 (0.11-2.21) | 0.3602 |
| Heart failure & cardiomyopathies | No | 139 (0.0028) | 1 (ref) | - | 1 (ref) | - |
|  | Yes | 12 (0.0206) | 2.73 (1.17-6.38) | 0.0207 | 1.83 (0.69-4.89) | 0.2263 |
| Ischemic heart disease | No | 139 (0.0028) | 1 (ref) | - | 1 (ref) | - |
|  | Yes | 12 (0.0111) | 1.82 (0.88-3.79) | 0.1088 | 1.07 (0.43-2.63) | 0.8907 |
| Hyperthyroidism | No | 147 (0.0030) | 1 (ref) | - | 1 (ref) | - |
|  | Yes | 4 (0.0100) | 2.08 (0.6-7.19) | 0.2480 | 2.04 (0.46-9.19) | 0.3507 |
| Obstructive sleep apnea | No | 143 (0.0029) | 1 (ref) | - | 1 (ref) | - |
|  | Yes | 8 (0.0082) | 2.18 (0.87-5.43) | 0.0946 | 1.7 (0.57-5.08) | 0.3396 |
| Anti-arrhythmics | No | 112 (0.0024) | 1 (ref) | - | 1 (ref) | - |
|  | Yes | 39 (0.0146) | 2.65 (1.59-4.41) | <0.001 | 3.03 (1.63-5.63) | <0.001 |
| Vaccination | No | 148 (0.0030) | 1 (ref) | - | 1 (ref) | - |
|  | Yes | 3 (0.0024) | 0.81 (0.21-3.16) | 0.7654 | 1.07 (0.22-5.25) | 0.9336 |

RR=Relative Risk. CI= Confidence Interval. wCCI=weighted Charlson Comorbidity Index.

Supplementary table 13: risk of first ever paroxysmal supraventricular tachycardia (PSVT) in the MCS stratified by pandemic wave.

| Wave | Variables | Levels | PSVT, n (%) | Univariable RR (95%CI) | p-value | Multivariable RR (95%CI) | p-value |
| --- | --- | --- | --- | --- | --- | --- | --- |
| 1^st^ | COVID-19 | Neg. | 13 (0.0058) | 1 (ref) | - | 1 (ref) | - |
|  |  | Pos. | 14 (0.0222) | 3.7 (1.73-7.92) | <0.001 | 3.88 (1.17-12.9) | 0.0269 |
|  | wCCI | 0 | 20 (0.0076) | 1 (ref) | - | 1 (ref) | - |
|  |  | 1-2 | 6 (0.0483) | 5.36 (1.51-19.05) | 0.0095 | 1.84 (0.26-12.86) | 0.5368 |
|  |  | 3-4 | 1 (0.0286) | 639416.81 (0-Inf) | 0.9769 | 39261.95 (0-inf.) | 0.9747 |
|  |  | >=5 | 0 (0.0000) | 0 (0-Inf) | 0.9845 | 0 (0-Inf) | 0.9806 |
|  | Heart failure & cardiomyopathies | No | 21 (0.0076) | 1 (ref) | - | 1 (ref) | - |
|  |  | Yes | 6 (0.0614) | 13.34 (1.55-114.92) | 0.0184 | 17.26 (1.41-210.51) | 0.0256 |
|  | Ischemic heart disease | No | 24 (0.0089) | 1 (ref) | - | 1 (ref) | - |
|  |  | Yes | 3 (0.0209) | 1.85 (0.39-8.88) | 0.4414 | 0.51 (0.03-7.98) | 0.6284 |
|  | Hyperthyroidism | No | 26 (0.0092) | 1 (ref) | - | 1 (ref) | - |
|  |  | Yes | 1 (0.0289) | 1.4 (0.12-16.4) | 0.7901 | 2.41 (0.07-87.15) | 0.6304 |
|  | Obstructive sleep apnea | No | 24 (0.0086) | 1 (ref) | - | 1 (ref) | - |
|  |  | Yes | 3 (0.0406) | 7.89 (0.79-79.17) | 0.0792 | 1.35 (0.08-21.8) | 0.8317 |
|  | Anti-arrhythmics | No | 19 (0.0075) | 1 (ref) | - | 1 (ref) | - |
|  |  | Yes | 8 (0.0261) | 2.41 (0.82-7.09) | 0.1095 | 1.61 (0.33-7.93) | 0.5601 |
| 2^nd^ | COVID-19 | Neg. | 32 (0.0022) | 1 (ref) | - | 1 (ref) | - |
|  |  | Pos. | 32 (0.0082) | 3.71 (2.27-6.07) | <0.001 | 3.6 (2.15-6.04) | <0.001 |
|  | wCCI | 0 | 57 (0.0037) | 1 (ref) | - | 1 (ref) | - |
|  |  | 1-2 | 4 (0.0022) | 0.51 (0.17-1.53) | 0.2321 | 0.73 (0.23-2.35) | 0.6012 |
|  |  | 3-4 | 0 (0.0000) | 0 (0-inf.) | 0.9504 | 0 (0-inf) | 0.9686 |
|  |  | >=5 | 3 (0.0038) | 0.74 (0.21-2.69) | 0.6505 | 0.81 (0.2-3.35) | 0.7760 |
|  | Heart failure & cardiomyopathies | No | 60 (0.0033) | 1 (ref) | - | 1 (ref) | - |
|  |  | Yes | 4 (0.0171) | 1.5 (0.42-5.4) | 0.5323 | 1.13 (0.26-4.94) | 0.8728 |
|  | Ischemic heart disease | No | 60 (0.0034) | 1 (ref) | - | 1 (ref) | - |
|  |  | Yes | 4 (0.0093) | 1.26 (0.41-3.91) | 0.6899 | 0.8 (0.2-3.1) | 0.7441 |
|  | Hyperthyroidism | No | 64 (0.0035) | 1 (ref) | - | 1 (ref) | - |
|  |  | Yes | 0 (0.0000) | 0 (0-inf.) | 0.9451 | 0 (0-inf.) | 0.9640 |
|  | Obstructive sleep apnea | No | 61 (0.0034) | 1 (ref) | - | 1 (ref) | - |
|  |  | Yes | 3 (0.0082) | 4 (0.81-19.82) | 0.0895 | 4.02 (0.69-23.31) | 0.1204 |
|  | Anti-arrhythmics | No | 45 (0.0026) | 1 (ref) | - | 1 (ref) | - |
|  |  | Yes | 19 (0.0182) | 2.43 (1.16-5.08) | 0.0181 | 3.14 (1.29-7.67) | 0.0120 |
| 3^rd^ | COVID-19 | Neg. | 20 (0.0009) | 1 (ref) | - | 1 (ref) | - |
|  |  | Pos. | 40 (0.0068) | 7.85 (4.59-13.43) | <0.001 | 8.59 (4.71-15.66) | <0.001 |
|  | wCCI | 0 | 60 (0.0021) | 1 (ref) | - | 1 (ref) | - |
|  |  | 1-2 | 0 (0.0000) | 1 (0-Inf) | 1.0000 | 1.07 (0-Inf) | 1.0000 |
|  |  | 3-4 | 0 (0.0000) | 1 (0-Inf) | 1.0000 | 1.01 (0-Inf) | 1.0000 |
|  |  | >=5 | 0 (0.0000) | 1 (0-Inf) | 1.0000 | 1.03 (0-Inf) | 1.0000 |
|  | Heart failure & cardiomyopathies | No | 58 (0.0020) | 1 (ref) | - | 1 (ref) | - |
|  |  | Yes | 2 (0.0080) | 1.77 (0.28-11.12) | 0.5449 | 0.52 (0.04-7.27) | 0.6287 |
|  | Ischemic heart disease | No | 55 (0.0019) | 1 (ref) | - | 1 (ref) | - |
|  |  | Yes | 5 (0.0099) | 3.17 (0.82-12.24) | 0.0936 | 1.23 (0.2-7.54) | 0.8244 |
|  | Hyperthyroidism | No | 57 (0.0020) | 1 (ref) | - | 1 (ref) | - |
|  |  | Yes | 3 (0.0142) | 298961.84 (0-inf.) | 0.9363 | 143301.95 (0-inf.) | 0.9513 |
|  | Obstructive sleep apnoea | No | 58 (0.0020) | 1 (ref) | - | 1 (ref) | - |
|  |  | Yes | 2 (0.0037) | 0.79 (0.16-3.79) | 0.7635 | 0.74 (0.1-5.54) | 0.7709 |
|  | Anti-arrhythmics | No | 48 (0.0017) | 1 (ref) | - | 1 (ref) | - |
|  |  | Yes | 12 (0.0091) | 3.25 (1.27-8.3) | 0.0136 | 4.19 (1.11-15.78) | 0.0344 |
|  | Vaccination | No | 57 (0.0021) | 1 (ref) | - | 1 (ref) | - |
|  |  | Yes | 3 (0.0024) | 0.81 (0.21-3.16) | 0.7654 | 1.63 (0.29-8.98) | 0.5764 |

Supplementary table 14: risk of first ever paroxysmal supraventricular tachycardia (PSVT) in the MCS stratified by disease severity (hospitalized and non-hospitalized individuals).

| COVID-19 severity | Variables | Levels | PSVT, n (%) | Univariable RR (95%CI) | p-value | Multivariable RR (95%CI) | p-value |
| --- | --- | --- | --- | --- | --- | --- | --- |
| Non-hospitalised | COVID-19 | Neg. | 62 (0.0016) | 1 (ref) | - | 1 (ref) | - |
|  |  | Pos. | 39 (0.0040) | 2.38 (1.6-3.56) | <0.001 | 2.61 (1.71-3.97) | <0.001 |
|  | wCCI | 0 | 94 (0.0021) | 1 (ref) | - | 1 (ref) | - |
|  |  | 1-2 | 4 (0.0020) | 0.59 (0.2-1.78) | 0.3509 | 0.45 (0.13-1.48) | 0.1856 |
|  |  | 3-4 | 0 (0.0000) | 0 (0-inf.) | 0.9484 | 0 (0-inf.) | 0.9668 |
|  |  | >=5 | 3 (0.0035) | 1.25 (0.33-4.78) | 0.7399 | 0.85 (0.19-3.77) | 0.8266 |
|  | Heart failure & cardiomyopathies | No | 95 (0.0020) | 1 (ref) | - | 1 (ref) | - |
|  |  | Yes | 6 (0.0140) | 4.92 (1.19-20.38) | 0.0282 | 3.79 (0.82-17.41) | 0.0873 |
|  | Ischemic heart disease | No | 96 (0.0020) | 1 (ref) | - | 1 (ref) | - |
|  |  | Yes | 5 (0.0060) | 1.83 (0.6-5.56) | 0.2847 | 1.38 (0.4-4.71) | 0.6073 |
|  | Hyperthyroidism | No | 99 (0.0021) | 1 (ref) | - | 1 (ref) | - |
|  |  | Yes | 2 (0.0054) | 2 (0.37-10.92) | 0.4235 | 1.44 (0.2-10.33) | 0.7168 |
|  | Obstructive sleep apnoea | No | 98 (0.0021) | 1 (ref) | - | 1 (ref) | - |
|  |  | Yes | 3 (0.0035) | 1.45 (0.35-6.04) | 0.6132 | 1.36 (0.26-6.97) | 0.7160 |
|  | Anti-arrhythmics | No | 79 (0.0017) | 1 (ref) | - | 1 (ref) | - |
|  |  | Yes | 22 (0.0100) | 3.29 (1.64-6.6) | <0.001 | 3.37 (1.57-7.2) | 0.0017 |
|  | Vaccination | No | 99 (0.0021) | 1 (ref) | - | 1 (ref) | - |
|  |  | Yes | 2 (0.0017) | 0.97 (0.19-4.94) | 0.9671 | 1.34 (0.26-7.05) | 0.7291 |
| Hospitalised | COVID-19 | Negative | 3 (0.0016) | 1 (ref) | - | 1 (ref) | - |
|  |  | Positive | 47 (0.0954) | 57.86 (17.99-186.09) | <0.001 | 205.48 (16.77-2517.34) | <0.001 |
|  | wCCI | 0 | 43 (0.0200) | 1 (ref) | - | 1 (ref) | - |
|  |  | 1-2 | 6 (0.0497) | 3.4 (1.08-10.68) | 0.0361 | 1.48 (0.02-90.29) | 0.8504 |
|  |  | 3-4 | 1 (0.0334) | 1.62 (0.14-19) | 0.7011 | 0.69 (0-inf.) | 0.9661 |
|  |  | >=5 | 0 (0.0000) | 0 (0-inf.) | 0.9559 | 0 (0-Inf) | 0.9746 |
|  | Heart failure & cardiomyopathies | No | 44 (0.0199) | 1 (ref) | - | 1 (ref) | - |
|  |  | Yes | 6 (0.0394) | 1.88 (0.62-5.68) | 0.2642 | 3.44 (0.2-60.54) | 0.3979 |
|  | Ischemic heart disease | No | 43 (0.0203) | 1 (ref) | - | 1 (ref) | - |
|  |  | Yes | 7 (0.0290) | 1.81 (0.68-4.82) | 0.2323 | 0.15 (0-4.56) | 0.2764 |
|  | Hyperthyroidism | No | 48 (0.0206) | 1 (ref) | - | 1 (ref) | - |
|  |  | Yes | 2 (0.0648) | 2.17 (0.35-13.47) | 0.4043 | 4.71 (0.01-2784.1) | 0.6340 |
|  | Obstructive sleep apnoea | No | 45 (0.0200) | 1 (ref) | - | 1 (ref) | - |
|  |  | Yes | 5 (0.0438) | 2.97 (0.9-9.86) | 0.0748 | 1.67 (0.06-48.02) | 0.7636 |
|  | Anti-arrhythmics | No | 33 (0.0174) | 1 (ref) | - | 1 (ref) | - |
|  |  | Yes | 17 (0.0364) | 2.05 (0.98-4.32) | 0.0582 | 11.17 (0.78-160.95) | 0.0763 |
|  | Vaccination | No | 49 (0.0215) | 1 (ref) | - | 1 (ref) | - |
|  |  | Yes | 1 (0.0123) | 0.58 (0.05-6.36) | 0.6575 | 0.69 (0-113205.4) | 0.9519 |

Supplementary table 15: Effect modification for bradyarrhythmias by sex (males and females) in the self-controlled case series study, presented by predefined times periods in days relative to COVID-19 date. IRR: Incidence Rate Ratio; CI: Confidence Intervals.

|  | Males | | | Females | | |
| --- | --- | --- | --- | --- | --- | --- |
| Period (days) | Events, No | IRR (95%CI) | P value | Events, No | IRR (95%CI) | P value |
| Control period | 319 | 1 (ref) |  | 155 | 1 (ref) |  |
| -30 to -4 | 55 | 1.69 (1.23 to 2.31) | 0.0011 | 49 | 3.29 (2.31 to 4.7) | <0.001 |
| -3 to -1 | 4 | 3.03 (1.12 to 8.22) | 0.0293 | 4 | 6.95 (2.53 to 19.08) | <0.001 |
| 0 | 20 | 15 (9.31 to 24.17) | <0.001 | 15 | 25.32 (14.43 to 44.41) | <0.001 |
| 1 to 7 | 29 | 4.01 (2.66 to 6.02) | <0.001 | 14 | 3.88 (2.17 to 6.94) | <0.001 |
| 8 to 14 | 18 | 2.8 (1.7 to 4.61) | <0.001 | 12 | 3.56 (1.91 to 6.65) | <0.001 |
| 15 to 30 | 23 | 1.65 (1.05 to 2.6) | 0.0286 | 5 | 0.7 (0.28 to 1.75) | 0.4479 |
| 31 to 60 | 34 | 1.47 (0.99 to 2.17) | 0.0543 | 15 | 1.11 (0.63 to 1.97) | 0.7158 |
| 61 to 90 | 17 | 0.8 (0.47 to 1.35) | 0.4046 | 13 | 1.09 (0.59 to 2.01) | 0.7928 |
| 91 to 180 | 39 | 0.89 (0.59 to 1.33) | 0.5605 | 33 | 1.36 (0.86 to 2.14) | 0.1907 |

Supplementary table 16: Effect modification for bradyarrhythmias by age (<=60 years, and >60 years) in the self-controlled case series study, presented by predefined times periods in days relative to COVID-19 date. IRR: Incidence Rate Ratio; CI: Confidence Intervals.

|  | <=60 | | | >60 | | |
| --- | --- | --- | --- | --- | --- | --- |
| Period (days) | Events, No | IRR (95%CI) | P value | Events, No | IRR (95%CI) | P value |
| Control period | 89 | 1 (ref) |  | 385 | 1 (ref) |  |
| -30 to -4 | 9 | 1.4 (0.68 to 2.88) | 0.3629 | 95 | 2.25 (1.75 to 2.89) | <0.001 |
| -3 to -1 | 0 | 0 (0 to Inf) | 0.9951 | 8 | 4.68 (2.3 to 9.55) | <0.001 |
| 0 | 5 | 20.65 (8.11 to 52.6) | <0.001 | 30 | 17.17 (11.57 to 25.48) | <0.001 |
| 1 to 7 | 4 | 2.8 (1 to 7.86) | <0.001 | 39 | 4.04 (2.83 to 5.75) | <0.001 |
| 8 to 14 | 9 | 6.38 (3.07 to 13.28) | <0.001 | 21 | 2.47 (1.56 to 3.91) | <0.001 |
| 15 to 30 | 9 | 2.63 (1.26 to 5.5) | 0.0100 | 19 | 1.05 (0.65 to 1.69) | 0.8488 |
| 31 to 60 | 7 | 1.13 (0.5 to 2.57) | 0.7742 | 42 | 1.38 (0.97 to 1.96) | 0.0717 |
| 61 to 90 | 6 | 0.96 (0.39 to 2.34) | 0.9274 | 24 | 0.87 (0.56 to 1.36) | 0.5358 |
| 91 to 180 | 16 | 1.26 (0.65 to 2.42) | 0.4960 | 56 | 0.99 (0.71 to 1.39) | 0.9527 |

Supplementary table 17: Effect modification for bradyarrhythmias by pandemic wave in the self-controlled case series study, presented by predefined times periods in days relative to COVID-19 date. Pandemic waves were defined as follows: first wave February 1 to July 31, 2020; second wave August 1, 2020, to January 31, 2021; and third wave February 1 to May 25, 2021. IRR: Incidence Rate Ratio; CI: Confidence Intervals.

|  | Wave 1 | | | Wave 2 | | | Wave 3 | | |
| --- | --- | --- | --- | --- | --- | --- | --- | --- | --- |
| Period (days) | Events, No | IRR (95%CI) | P value | Events, No | IRR (95%CI) | P value | Events, No | IRR (95%CI) | P value |
| Control period | 46 | 1 (ref) |  | 249 | 1 (ref) |  | 179 | 1 (ref) |  |
| -30 to -4 | 26 | 6.37 (2.99 to 13.58) | <0.001 | 55 | 1.79 (1.21 to 2.65) | 0.0033 | 24* | 1.02 (0.56 to 1.85) | 0.9476 |
| -3 to -1 | 4 | 24.68 (7.41 to 82.23) | <0.001 | 3 | 2.5 (0.77 to 8.15) | 0.1275 |  |  |  |
| 0 | 8 | 48.32 (18.08 to 129.11) | <0.001 | 15 | 12.31 (6.69 to 22.65) | <0.001 | 12 | 13.51 (6.29 to 29.01) | <0.001 |
| 1 to 7 | 14 | 15.53 (6.47 to 37.27) | <0.001 | 21 | 2.99 (1.72 to 5.19) | <0.001 | 8 | 1.52 (0.64 to 3.65) | 0.3455 |
| 8 to 14 | 3 | 3.37 (0.88 to 12.86) | 0.0755 | 19 | 2.94 (1.64 to 5.25) | 0.0003 | 8 | 1.72 (0.7 to 4.22) | 0.2352 |
| 15 to 30 | 8 | 3.73 (1.38 to 10.06) | 0.0093 | 13 | 0.91 (0.47 to 1.8) | 0.7955 | 7 | 0.66 (0.25 to 1.75) | 0.4074 |
| 31 to 60 | 6 | 1.52 (0.52 to 4.41) | 0.4418 | 26 | 0.91 (0.5 to 1.65) | 0.7471 | 17 | 1.22 (0.51 to 2.93) | 0.6558 |
| 61 to 90 | 7 | 1.66 (0.6 to 4.59) | 0.3326 | 21 | 0.7 (0.35 to 1.38) | 0.3042 | 3** | 0.3 (0.07 to 1.37) | 0.1213 |
| 91 to 180 | 29 | 1.79 (0.86 to 3.7) | 0.1172 | 42 | 0.69 (0.36 to 1.35) | 0.2819 |  |  |  |

* Buffer period from -30 to -1 days; ** risk period from 61 to 180 days

Supplementary table 18: Effect modification for bradyarrhythmias by COVID-19 severity (non-hospitalised and hospitalised) in the self-controlled case series study, presented by predefined times periods in days relative to COVID-19 date. IRR: Incidence Rate Ratio; CI: Confidence Intervals.

|  | Not hospitalized | | | Hospitalized | | |
| --- | --- | --- | --- | --- | --- | --- |
| Period (days) | Events, No | IRR (95%CI) | P value | Events, No | IRR (95%CI) | P value |
| Control period | 295 | 1 (ref) |  | 179 | 1 (ref) |  |
| -30 to -4 | 31* | 1.08 (0.73 to 1.61) | 0.7028 | 74 | 3.77 (2.79 to 5.08) | <0.001 |
| -3 to -1 |  |  |  | 7 | 8.74 (4.05 to 18.89) | <0.001 |
| 0 | 6 | 5.99 (2.62 to 13.67) | <0.001 | 29 | 35.15 (23.04 to 53.63) | <0.001 |
| 1 to 7 | 7** | 0.56 (0.26 to 1.22) | 0.1434 | 41 | 9.15 (6.3 to 13.28) | <0.001 |
| 8 to 14 |  |  |  | 25 | 6.31 (4.03 to 9.89) | <0.001 |
| 15 to 30 | 10 | 0.77 (0.4 to 1.49) | 0.4427 | 18 | 2.13 (1.28 to 3.55) | 0.0036 |
| 31 to 60 | 24 | 1.06 (0.66 to 1.69) | 0.8140 | 25 | 1.69 (1.08 to 2.65) | 0.0229 |
| 61 to 90 | 19 | 0.93 (0.55 to 1.58) | 0.7990 | 11 | 0.76 (0.4 to 1.43) | 0.3884 |
| 91 to 180 | 46 | 1.21 (0.8 to 1.84) | 0.3715 | 26 | 0.79 (0.5 to 1.25) | 0.3056 |

* Buffer period from -30 to -1 days; ** risk period from 1 to 14 days

Supplementary table 19: Unadjusted and adjusted conditional Poisson regression models for first bradyarrhythmia event within 30 days after COVID-19 in matched cohort study.

| Variables | Levels | Bradyarrhythmia, n (%) | Univariable RR  (95%CI) | p-value | Multivariable RR (95%CI) | p-value |
| --- | --- | --- | --- | --- | --- | --- |
| COVID-19 | Neg. | 94 (0.0024) | 1 (ref) | - | 1 (ref) | - |
|  | Pos. | 101 (0.0097) | 3.99 (3.01-5.28) | <0.001 | 3.36 (2.42-4.68) | <0.001 |
| wCCI | 0 | 172 (0.0037) | 1 (ref) | - | 1 (ref) | - |
|  | 1-2 | 9 (0.0042) | 0.77 (0.36-1.63) | 0.4876 | 0.74 (0.31-1.78) | 0.5006 |
|  | 3-4 | 8 (0.0238) | 4.72 (1.63-13.64) | 0.0041 | 1.12 (0.28-4.56) | 0.8701 |
|  | >=5 | 6 (0.0065) | 1.42 (0.55-3.67) | 0.4695 | 0.4 (0.11-1.41) | 0.1528 |
| Heart failure & cardiomyopathies | No | 128 (0.0026) | 1 (ref) | - | 1 (ref) | - |
|  | Yes | 67 (0.1268) | 7.59 (4.79-12.03) | <0.001 | 5.02 (2.87-8.79) | <0.001 |
| Ischemic heart disease | No | 121 (0.0025) | 1 (ref) | - | 1 (ref) | - |
|  | Yes | 74 (0.0717) | 4.2 (2.83-6.24) | <0.001 | 2.6 (1.61-4.2) | <0.001 |
| Hyperthyroidism | No | 188 (0.0038) | 1 (ref) | - | 1 (ref) | - |
|  | Yes | 7 (0.0174) | 3.52 (1.22-10.12) | 0.0196 | 2.26 (0.64-8.02) | 0.2085 |
| Obstructive sleep apnea | No | 181 (0.0037) | 1 (ref) | - | 1 (ref) | - |
|  | Yes | 14 (0.0143) | 1.48 (0.77-2.85) | 0.2350 | 1.29 (0.58-2.88) | 0.5328 |
| Anti-arrhythmics | No | 120 (0.0025) | 1 (ref) | - | 1 (ref) | - |
|  | Yes | 75 (0.0285) | 1.34 (0.95-1.89) | 0.0907 | 0.75 (0.48-1.18) | 0.2128 |
| Vaccination | No | 188 (0.0038) | 1 (ref) | - | 1 (ref) | - |
|  | Yes | 7 (0.0055) | 0.83 (0.27-2.54) | 0.7406 | 1.62 (0.44-5.98) | 0.4700 |

RR=Relative Risk. CI= Confidence Interval. wCCI=weighted Charlson Comorbidity Index.

Supplementary table 20: risk of first ever bradyarrhythmia in the MCS stratified by pandemic wave.

| Wave | Variables | Levels | Bradyarrhythmia, n (%) | Univariable RR (95%CI) | p-value | Multivariable RR (95%CI) | p-value |
| --- | --- | --- | --- | --- | --- | --- | --- |
| 1^st^ | COVID-19 | Neg. | 21 (0.0095) | 1 (ref) | - | 1 (ref) | - |
|  |  | Pos. | 24 (0.0383) | 4.05 (2.25-7.29) | <0.001 | 5.65 (1.84-17.31) | 0.0024 |
|  | wCCI | 0 | 30 (0.0115) | 1 (ref) | - | 1 (ref) | - |
|  |  | 1-2 | 5 (0.0404) | 3.67 (1.06-12.71) | 0.0402 | 0.9 (0.13-6.14) | 0.9102 |
|  |  | 3-4 | 7 (0.2067) | 12 (2.49-57.93) | 0.0020 | 1.65 (0.18-15.5) | 0.6606 |
|  |  | >=5 | 3 (0.0467) | 1.81 (0.45-7.27) | 0.4016 | 0.09 (0.01-0.93) | 0.0435 |
|  | Heart failure & cardiomyopathies | No | 26 (0.0094) | 1 (ref) | - | 1 (ref) | - |
|  |  | Yes | 19 (0.2224) | 9.67 (3.82-24.43) | <0.001 | 10.47 (3.06-35.75) | <0.001 |
|  | Ischemic heart disease | No | 27 (0.0100) | 1 (ref) | - | 1 (ref) | - |
|  |  | Yes | 18 (0.1358) | 4.35 (1.94-9.8) | <0.001 | 2.28 (0.79-6.6) | 0.1286 |
|  | Hyperthyroidism | No | 43 (0.0153) | 1 (ref) | - | 1 (ref) | - |
|  |  | Yes | 2 (0.0584) | 6.62 (0.6-73.42) | 0.1235 | 2.13 (0.04-115.74) | 0.7098 |
|  | Obstructive sleep apnea | No | 45 (0.0163) | 1 (ref) | - | 1 (ref) | - |
|  |  | Yes | 0 (0.0000) | 0 (0-inf.) | 0.9484 | 0 (0-inf.) | 0.9626 |
|  | Anti-arrhythmics | No | 30 (0.0118) | 1 (ref) | - | 1 (ref) | - |
|  |  | Yes | 15 (0.0511) | 0.93 (0.46-1.9) | 0.8421 | 0.6 (0.2-1.78) | 0.3572 |
| 2^nd^ | COVID-19 | Neg. | 35 (0.0024) | 1 (ref) | - | 1 (ref) | - |
|  |  | Pos. | 43 (0.0110) | 4.46 (2.85-6.98) | <0.001 | 3.35 (2.02-5.57) | <0.001 |
|  | wCCI | 0 | 70 (0.0046) | 1 (ref) | - | 1 (ref) | - |
|  |  | 1-2 | 4 (0.0022) | 0.36 (0.12-1.04) | 0.0583 | 0.62 (0.19-2.05) | 0.4358 |
|  |  | 3-4 | 1 (0.0036) | 0.94 (0.1-8.43) | 0.9562 | 0.13 (0.01-2.06) | 0.1494 |
|  |  | >=5 | 3 (0.0038) | 1.09 (0.29-4.05) | 0.8991 | 1.02 (0.18-5.94) | 0.9812 |
|  | Heart failure & cardiomyopathies | No | 54 (0.0030) | 1 (ref) | - | 1 (ref) | - |
|  |  | Yes | 24 (0.1142) | 6.06 (2.92-12.56) | <0.001 | 4.06 (1.49-11.07) | 0.0062 |
|  | Ischemic heart disease | No | 48 (0.0027) | 1 (ref) | - | 1 (ref) | - |
|  |  | Yes | 30 (0.0733) | 4.8 (2.48-9.29) | <0.001 | 3.49 (1.57-7.77) | 0.0022 |
|  | Hyperthyroidism | No | 75 (0.0041) | 1 (ref) | - | 1 (ref) | - |
|  |  | Yes | 3 (0.0193) | 2.5 (0.55-11.4) | 0.2378 | 1.51 (0.22-10.32) | 0.6771 |
|  | Obstructive sleep apnea | No | 71 (0.0040) | 1 (ref) | - | 1 (ref) | - |
|  |  | Yes | 7 (0.0193) | 1.88 (0.73-4.82) | 0.1903 | 1.13 (0.32-3.99) | 0.8496 |
|  | Anti-arrhythmics | No | 53 (0.0031) | 1 (ref) | - | 1 (ref) | - |
|  |  | Yes | 25 (0.0243) | 0.97 (0.56-1.67) | 0.9156 | 0.53 (0.25-1.12) | 0.0977 |
| 3^rd^ | COVID-19 | Neg. | 38 (0.0016) | 1 (ref) | - | 1 (ref) | - |
|  |  | Pos. | 34 (0.0058) | 3.49 (2.2-5.55) | <0.001 | 3.14 (1.83-5.39) | <0.001 |
|  | wCCI | 0 | 72 (0.0025) | 1 (ref) | - | 1 (ref) | - |
|  |  | 1-2 | 0 (0.0000) | 1 (0-Inf) | 1.0000 | 1.17 (0-Inf) | 1.0000 |
|  |  | 3-4 | 0 (0.0000) | 1 (0-Inf) | 1.0000 | 1.06 (0-Inf) | 1.0000 |
|  |  | >=5 | 0 (0.0000) | 1 (0-Inf) | 1.0000 | 1.13 (0-Inf) | 1.0000 |
|  | Heart failure & cardiomyopathies | No | 48 (0.0017) | 1 (ref) | - | 1 (ref) | - |
|  |  | Yes | 24 (0.1031) | 8.1 (3.71-17.68) | <0.001 | 4.94 (1.97-12.4) | <0.001 |
|  | Ischemic heart disease | No | 46 (0.0016) | 1 (ref) | - | 1 (ref) | - |
|  |  | Yes | 26 (0.0531) | 3.63 (1.94-6.81) | <0.001 | 2.2 (1.03-4.72) | 0.0420 |
|  | Hyperthyroidism | No | 70 (0.0024) | 1 (ref) | - | 1 (ref) | - |
|  |  | Yes | 2 (0.0094) | 4 (0.56-28.4) | 0.1657 | 3.92 (0.4-38.66) | 0.2428 |
|  | Obstructive sleep apnea | No | 65 (0.0023) | 1 (ref) | - | 1 (ref) | - |
|  |  | Yes | 7 (0.0129) | 2.31 (0.85-6.31) | 0.1023 | 2.46 (0.83-7.3) | 0.1049 |
|  | Anti-arrhythmics | No | 37 (0.0013) | 1 (ref) | - | 1 (ref) | - |
|  |  | Yes | 35 (0.0267) | 2.51 (1.39-4.54) | 0.0023 | 1.48 (0.72-3.05) | 0.2913 |
|  | Vaccination | No | 65 (0.0023) | 1 (ref) | - | 1 (ref) | - |
|  |  | Yes | 7 (0.0055) | 0.83 (0.27-2.54) | 0.7406 | 1.42 (0.38-5.31) | 0.6034 |

|  |  |  |  |  |  |  |  |
| --- | --- | --- | --- | --- | --- | --- | --- |
|  |  |  |  |  |  |  |  |
|  |  |  |  |  |  |  |  |
|  |  |  |  |  |  |  |  |
|  |  |  |  |  |  |  |  |
|  |  |  |  |  |  |  |  |
|  |  |  |  |  |  |  |  |
|  |  |  |  |  |  |  |  |
|  |  |  |  |  |  |  |  |
|  |  |  |  |  |  |  |  |
|  |  |  |  |  |  |  |  |

Supplementary table 21: risk of first ever bradyarrhythmia in the MCS stratified by disease severity (hospitalized and non-hospitalized individuals).

| COVID-19 severity | Variables | Levels | Bradyarrhythmia, n (%) | Univariable RR (95%CI) | p-value | Multivariable RR (95%CI) | p-value |
| --- | --- | --- | --- | --- | --- | --- | --- |
| Non-hospitalised | COVID-19 | Neg. | 69 (0.0018) | 1 (ref) | - | 1 (ref) | - |
|  |  | Pos. | 17 (0.0017) | 0.92 (0.54-1.56) | 0.7543 | 0.8 (0.44-1.43) | 0.4477 |
|  | wCCI | 0 | 79 (0.0018) | 1 (ref) | - | 1 (ref) | - |
|  |  | 1-2 | 5 (0.0025) | 1.43 (0.47-4.3) | 0.5276 | 1.52 (0.46-5.02) | 0.4899 |
|  |  | 3-4 | 0 (0.0000) | 0 (0-inf.) | 0.9556 | 0 (0-inf.) | 0.9441 |
|  |  | >=5 | 2 (0.0023) | 1.08 (0.22-5.23) | 0.9259 | 1.31 (0.23-7.29) | 0.7610 |
|  | Heart failure & cardiomyopathies | No | 65 (0.0014) | 1 (ref) | - | 1 (ref) | - |
|  |  | Yes | 21 (0.0531) | 4.5 (2.26-8.97) | <0.001 | 4.54 (2.09-9.87) | <0.001 |
|  | Ischemic heart disease | No | 62 (0.0013) | 1 (ref) | - | 1 (ref) | - |
|  |  | Yes | 24 (0.0296) | 2.48 (1.35-4.56) | 0.0035 | 2.01 (1.03-3.91) | 0.0399 |
|  | Hyperthyroidism | No | 83 (0.0017) | 1 (ref) | - | 1 (ref) | - |
|  |  | Yes | 3 (0.0080) | 2.5 (0.55-11.4) | 0.2378 | 1.84 (0.36-9.39) | 0.4610 |
|  | Obstructive sleep apnea | No | 83 (0.0018) | 1 (ref) | - | 1 (ref) | - |
|  |  | Yes | 3 (0.0035) | 0.71 (0.2-2.53) | 0.5960 | 0.76 (0.2-2.85) | 0.6789 |
|  | Anti-arrhythmics | No | 52 (0.0011) | 1 (ref) | - | 1 (ref) | - |
|  |  | Yes | 34 (0.0155) | 1.47 (0.87-2.48) | 0.1552 | 0.92 (0.5-1.7) | 0.7957 |
|  | Vaccination | No | 81 (0.0017) | 1 (ref) | - | 1 (ref) | - |
|  |  | Yes | 5 (0.0042) | 1.24 (0.3-5.08) | 0.7683 | 1.19 (0.24-5.85) | 0.8341 |
| Hospitalised | COVID-19 | Negative | 25 (0.0137) | 1 (ref) | - | 1 (ref) | - |
|  |  | Positive | 84 (0.1728) | 12.41 (7.93-19.41) | <0.001 | 14.26 (7.66-26.52) | <0.001 |
|  | wCCI | 0 | 93 (0.0440) | 1 (ref) | - | 1 (ref) | - |
|  |  | 1-2 | 4 (0.0339) | 0.48 (0.16-1.42) | 0.1844 | 0.34 (0.07-1.73) | 0.1959 |
|  |  | 3-4 | 8 (0.2756) | 9.4 (2.49-35.54) | 0.0010 | 1.21 (0.15-9.62) | 0.8584 |
|  |  | >=5 | 4 (0.0691) | 1.75 (0.53-5.83) | 0.3616 | 0.07 (0.01-0.63) | 0.0174 |
|  | Heart failure & cardiomyopathies | No | 63 (0.0288) | 1 (ref) | - | 1 (ref) | - |
|  |  | Yes | 46 (0.3464) | 11.17 (5.87-21.29) | <0.001 | 6.59 (2.33-18.62) | <0.001 |
|  | Ischemic heart disease | No | 59 (0.0281) | 1 (ref) | - | 1 (ref) | - |
|  |  | Yes | 50 (0.2260) | 6.15 (3.59-10.54) | <0.001 | 3.63 (1.52-8.68) | 0.0038 |
|  | Hyperthyroidism | No | 105 (0.0459) | 1 (ref) | - | 1 (ref) | - |
|  |  | Yes | 4 (0.1332) | 4.92 (1.1-22.05) | 0.0374 | 2.32 (0.08-64.02) | 0.6197 |
|  | Obstructive sleep apnea | No | 98 (0.0444) | 1 (ref) | - | 1 (ref) | - |
|  |  | Yes | 11 (0.0998) | 2.11 (0.97-4.6) | 0.0598 | 2.09 (0.51-8.59) | 0.3079 |
|  | Anti-arrhythmics | No | 68 (0.0362) | 1 (ref) | - | 1 (ref) | - |
|  |  | Yes | 41 (0.0929) | 1.26 (0.8-1.97) | 0.3132 | 0.84 (0.38-1.85) | 0.6612 |
|  | Vaccination | No | 107 (0.0478) | 1 (ref) | - | 1 (ref) | - |
|  |  | Yes | 2 (0.0257) | 0.42 (0.05-3.48) | 0.4184 | 4.11 (0.43-39.43) | 0.2203 |

Supplementary table 22: Incidence Rate Ratio (IRR) with 95% confidence intervals (CI) of a first ventricular arrhythmia event following COVID-19 in the self-controlled case series (SCCS) study, presented by predefined times periods in days relative to COVID-19 date.

| Period (days) | Events, No | IRR (95%CI) | P value |
| --- | --- | --- | --- |
| Control period | 65 | 1 (ref) |  |
| -30 to -4 | 9 | 1.75 (0.82 to 3.71) | 0.1469 |
| -3 to -1 | 0 | 0 (0 to Inf) | 0.9953 |
| 0 | 4 | 16.33 (5.52 to 48.32) | <0.001 |
| 1 to 14 | 6 | 2.04 (0.81 to 5.14) | 0.1289 |
| 15 to 30 | 3 | 1.27 (0.38 to 4.28) | 0.7016 |
| 31 to 60 | 7 | 1.69 (0.69 to 4.13) | 0.2495 |
| 61 to 90 | 3 | 0.89 (0.26 to 3.12) | 0.8615 |
| 91 to 180 | 4 | 0.59 (0.19 to 1.82) | 0.3579 |
|  |  |  |  |
| 1 to 30 | 6 | 1.66 (0.76 to 3.64) | 0.2060 |

Supplementary table 23: Unadjusted and adjusted conditional Poisson regression models for first ventricular arrhythmia event within 30 days after COVID-19 in matched cohort study.

| Variables | Levels | Ventricular arrhythmia, n (%) | Univariable RR  (95%CI) | p-value | Multivariable RR (95%CI) | p-value |
| --- | --- | --- | --- | --- | --- | --- |
| COVID-19 | Neg. | 28 (0.0007) | 1 (ref) | - | 1 (ref) | - |
|  | Pos. | 9 (0.0009) | 1.2 (0.57-2.55) | 0.6331 | 0.76 (0.17-3.35) | 0.7172 |
| wCCI | 0 | 30 (0.0006) | 1 (ref) | - | 1 (ref) | - |
|  | 1-2 | 4 (0.0019) | 1.65 (0.45-6.07) | 0.4510 | 2.59 (0.36-18.67) | 0.3443 |
|  | 3-4 | 2 (0.0059) | 6.47 (0.57-73.25) | 0.1314 | 13.04 (0-31582446.41) | 0.7321 |
|  | >=5 | 1 (0.0011) | 4 (0.25-63.95) | 0.3270 | 0.03 (0-1.96) | 0.1025 |
| Heart failure & cardiomyopathies | No | 22 (0.0004) | 1 (ref) | - | 1 (ref) | - |
|  | Yes | 15 (0.0252) | 48.38 (6.34-368.99) | <0.001 | 24.65 (1.47-413.46) | 0.0259 |
| Ischemic heart disease | No | 15 (0.0003) | 1 (ref) | - | 1 (ref) | - |
|  | Yes | 22 (0.0200) | 71.89 (9.65-535.71) | <0.001 | 76.28 (5.11-1137.67) | 0.0017 |
| Hyperthyroidism | No | 37 (0.0007) | 1 (ref) | - | 1 (ref) | - |
|  | Yes | 0 (0.0000) | 0 (0-inf.) | 0.9753 | 0 (0-Inf) | 0.9883 |
| Obstructive sleep apnea | No | 35 (0.0007) | 1 (ref) | - | 1 (ref) | - |
|  | Yes | 2 (0.0020) | 1.36 (0.26-7.2) | 0.7160 | 0.94 (0.03-29.79) | 0.9709 |
| Anti-arrhythmics | No | 21 (0.0004) | 1 (ref) | - | 1 (ref) | - |
|  | Yes | 16 (0.0058) | 5.58 (2.32-13.42) | <0.001 | 4.09 (0.96-17.44) | 0.0571 |
| Vaccination | No | 36 (0.0007) | 1 (ref) | - | 1 (ref) | - |
|  | Yes | 1 (0.0008) | 26392.36 (0-inf.) | 0.9750 | 16113.39 (0-Inf) | 0.9854 |

RR=Relative Risk. CI= Confidence Interval. wCCI=weighted Charlson Comorbidity Index.
