# Supplementary material for: Risk of arrhythmias following COVID-19: nationwide self-controlled case series and matched cohort study
Source: Eur Heart J Open. 2023 Nov 21;3(6):oead120. doi: 10.1093/ehjopen/oead120 (PMC10711544; doi:10.1093/ehjopen/oead120)
Supplement: oead120_Supplementary_Data [file oead120_supplementary_data.zip › Suplemental Publication Material (Figures).pptx]

## Slide 1
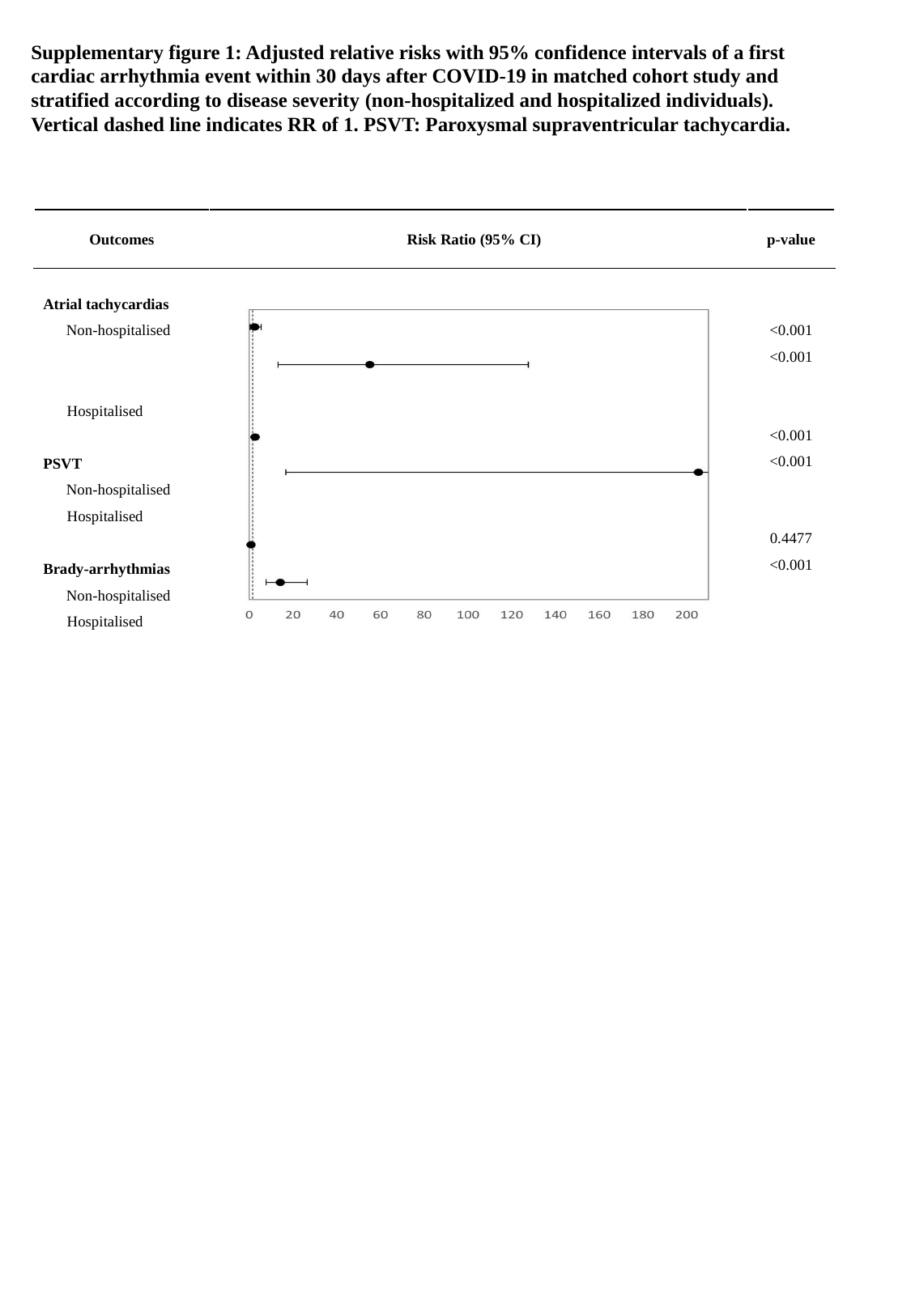

Supplementary figure 1: Adjusted relative risks with 95% confidence intervals of a first cardiac arrhythmia event within 30 days after COVID-19 in matched cohort study and stratified according to disease severity (non-hospitalized and hospitalized individuals). Vertical dashed line indicates RR of 1. PSVT: Paroxysmal supraventricular tachycardia.
| Outcomes | Risk Ratio (95% CI) | p-value |
| --- | --- | --- |
| Atrial tachycardias Non-hospitalised Hospitalised   PSVT Non-hospitalised Hospitalised   Brady-arrhythmias Non-hospitalised Hospitalised | | <0.001 <0.001 |
| | | <0.001 <0.001 |
| | | 0.4477 <0.001 |

## Slide 2
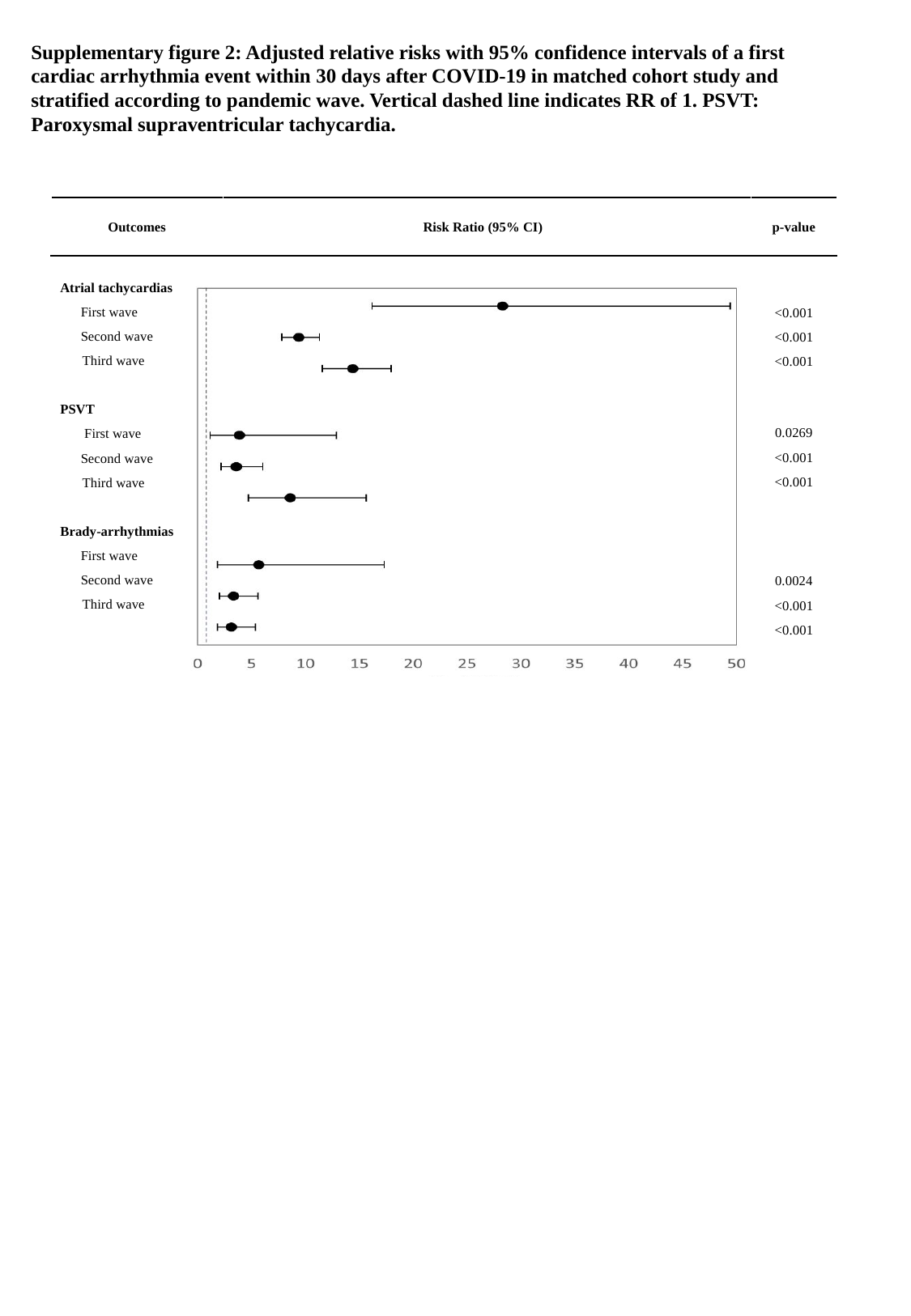

Supplementary figure 2: Adjusted relative risks with 95% confidence intervals of a first cardiac arrhythmia event within 30 days after COVID-19 in matched cohort study and stratified according to pandemic wave. Vertical dashed line indicates RR of 1. PSVT: Paroxysmal supraventricular tachycardia.
| Outcomes | Risk Ratio (95% CI) | p-value |
| --- | --- | --- |
| Atrial tachycardias First wave Second wave Third wave   PSVT First wave Second wave Third wave   Brady-arrhythmias First wave Second wave Third wave | | <0.001 <0.001 <0.001 |
| | | 0.0269 <0.001 <0.001 |
| | | 0.0024 <0.001 <0.001 |
